# Prevalence and clinical correlates of *Gardnerella* spp., *Fannyhessea vaginae*, *Lactobacillus crispatus* and *L. iners* in pregnant women in Bukavu, Democratic Republic of the Congo

**DOI:** 10.3389/fcimb.2024.1514884

**Published:** 2025-01-17

**Authors:** Lisa Himschoot, Guy Mulinganya, Tess Rogier, Ghislain Bisimwa, Freddy Kampara, Yvette Kujirakwinja, Jules Mongane, Innocent Mubalama, Steven Callens, Mario Vaneechoutte, Piet Cools

**Affiliations:** ^1^ Department of Diagnostic Sciences, Faculty of Medicine and Health Sciences, Ghent University, Ghent, Belgium; ^2^ Faculty of Medicine, Catholic University of Bukavu, Bukavu, Democratic Republic of Congo; ^3^ Department of Obstetrics and Gynecology, Hôpital Provincial Général de Référence de Bukavu, Bukavu, Democratic Republic of Congo; ^4^ Department of Internal Medicine and Pediatrics, Faculty of Medicine and Health Sciences, Ghent University, Ghent, Belgium

**Keywords:** bacterial vaginosis, *Gardnerella*, molecular diagnosis, preterm birth, low birthweight, Democratic Republic of the Congo

## Abstract

**Background:**

*Gardnerella* is a key pathogen in bacterial vaginosis (BV), but the role of the different *Gardnerella* species remains unclear. We investigated the role of four *Gardnerella* species, as well as *Fannyhessea vaginae*, *Lactobacillus crispatus* and *L. iners* in BV.

**Methods:**

From 331 pregnant women from the Democratic Republic of the Congo, BV was diagnosed using Nugent scoring and a cervicovaginal lavage was used to quantify *G. leopoldii*, *G. piotii*, *G. swidsinskii*, *G. vaginalis, F. vaginae, L. crispatus and L. iners* by qPCR. Univariate associations between these species and clinical outcomes were assessed. A logistic regression model and ROC curves were calculated to determine the best diagnostic marker for BV.

**Results:**

Here, *L. iners* (75.8%) was the most prevalent species and *G. vaginalis* (36.0%) the most common *Gardnerella* species. All investigated *Gardnerella* spp. were prevalent (50.9-57.9%) in women with (asymptomatic) BV. Univariate analysis revealed no significant associations with clinical symptoms of BV, while *F. vaginae* (positive Whiff test, high pH), *G. vaginalis* (high pH) and *L. crispatus* (low pH) were associated with signs of BV. *G. piotii* was associated with markers of urinary tract infection. Women with *L. iners* had higher odds of delivering preterm. ROC analyses showed that *F. vaginae* was the best marker for BV (AUC 0.81), and the combined model further increased the diagnostic performance (AUC 0.90).

**Conclusion:**

All *Gardnerella* species were involved in BV, although none were associated with the most important clinical symptoms of BV and none emerged as a superior molecular marker for BV.

## Introduction

Bacterial vaginosis (BV) is the most common gynecological condition among women of reproductive age worldwide (Allsworth and Peipert, 2007), with a prevalence among the general population of approximately 25% both globally (Peebles et al., 2019) and in sub-Saharan Africa (Park et al., 2024). Besides being a discomforting and often recurrent/chronic condition, BV is also considered a significant risk factor for adverse pregnancy outcomes such as preterm birth (PTB) and low birthweight (LBW) (Hillier et al., 1995). Furthermore, BV has been associated with an increased risk for the acquisition of sexually transmitted infections, such as human immunodeficiency virus (HIV) (Atashili et al., 2008), herpes simplex virus-2 (HSV-2) (Cherpes et al., 2005), *Trichomonas vaginalis*, *Neisseria gonorrhoeae* and *Chlamydia trachomatis* (Brotman et al., 2010).

Microbiologically, BV is characterized by a dysbiosis of the healthy vaginal microbiome (VMB) (Muzny et al., 2019), normally characterized by the predominance of only a single *Lactobacillus* species, mostly *L. crispatus* or *L. iners* (Vaneechoutte, 2017a). The vaginal microbiome can be categorized into community-state types (CSTs), with CST-I representing a vaginal microbiome dominated by *L. crispatus*, CSTI-II dominated by *L. gasseri*, CST-III dominated by *L. iners*, CST-IV representing a diverse microbiome and finally CST-V dominated by *L. jensenii* (Ravel et al., 2011). The lactobacilli-dominated environment has a low pH due to production of lactic acid by lactobacilli, making the vagina a hostile environment for many pathogens, although it is generally known that *L. iners* is less protective than *L. crispatus* (Vaneechoutte, 2017a). In BV, the VMB is replaced by a polymicrobial VMB with *Gardnerella* as the key marker (Muzny et al., 2018). However, also *L. iners* has been found among women with BV (Vaneechoutte, 2017b). Additionally, *Gardnerella* has also been isolated from healthy women in several studies (Hickey and Forney, 2014). *G. vaginalis* was long considered the single species within the *Gardnerella* genus. This is because the 16S rRNA gene, which forms the basis for bacterial taxonomy and microbiome studies (Clarridge, 2004), lacks resolution needed to distinguish the different *Gardnerella* species (Vaneechoutte et al., 2019). In 2019, Vaneechoutte and coworkers showed the existence of minimum thirteen genomospecies in the genus (Vaneechoutte et al., 2019), of which six have been named validly: *Gardnerella greenwoodii*, *G. leopoldii*, *G. pickettii*, *G. piotii*, *G. swidsinskii* and *G. vaginalis* (Vaneechoutte et al., 2019; Sousa et al., 2023).

*Gardnerella* species mostly cooccur in the VMB rather than being present as a single species (Hill and Albert, 2019; Munch et al., 2024; Schuster et al., 2024) and their clinical relevance has been under investigation for several years. In non-pregnant reproductive-aged Canadian women, Hill and coworkers (2019) already demonstrated, using a cpn60 sequencing approach, that *G. vaginalis* and *G. swidsinskii*, but not *G. leopoldii* and *G. piotii*, were associated with vaginal discharge and malodor, typical symptoms of BV (Hill and Albert, 2019). Turner and coworkers showed that, in American women with recurrent BV, persistently high concentrations of genomospecies 12 (as defined by Vaneechoutte and colleagues (Vaneechoutte et al., 2019)) were associated with refractory responses after metronidazole treatment of BV, and persistently low concentrations of genomospecies 12 and *G. swidsinskii*/*G. leopoldii* with remission (Turner et al., 2021). Munch and coworkers recently documented that American non-pregnant BV-negative women colonized with three or more *Gardnerella* species had higher chance for incident BV within 100 days compared to women colonized with fewer *Gardnerella* species (Munch et al., 2024). In a cohort of pregnant women at high risk of recurrent preterm birth, *G. leopoldii*, but not *G. piotii*, *G. swidsinskii* or *G. vaginalis*, was associated with spontaneous preterm birth (Schuster et al., 2024).

However, the different *Gardnerella* spp. have not yet been studied in African women, known to harbor a VMB that can differ significantly compared to Caucasian women (Fettweis et al., 2014). Therefore, we aimed to investigate the distribution of *G. leopoldii*, *G. piotii*, *G. swidsinskii* and *G. vaginalis* and the correlates with clinical signs and symptoms and adverse pregnancy outcomes using species-specific quantitative PCR (qPCR) assays in a population of pregnant women from the Democratic Republic of the Congo.

## Materials and methods

### Ethical approval

Ethical approval was obtained by the Internal Review Board of the Catholic University of Bukavu (reference number UCB/CIE/NC/016/2016), the Ministry of Public Health (reference number 062/CD/DPS/SK/2017) and the Ethical Committee of Ghent University Hospital (reference number PA2014/003). All pregnant women that participated in this study signed an informed consent form.

### Study design and population

This research was part of the AVEONS (acronym for Angamiza Vizuri (Swahili for ‘stop’) Early Onset Neonatal Sepsis) study. The AVEONS project had the overall aim to study the prevalence and clinical correlates of vaginal infections in a population of pregnant women from Bukavu, Democratic Republic of the Congo (DRC). The prevalence (26.3%), risk factors and adverse pregnancy outcomes of second trimester BV [assessed by microscopy (Nugent score)], as well as the study design and population have been described elsewhere (Mulinganya et al., 2021). Briefly, the AVEONS study was a prospective observational study where pregnant women were seen between 16 and 20 weeks of gestation [visit 1 (V1)], between 36 and 38 weeks (V2) and at delivery. Participants were recruited from January to October 2017 at the Provincial Referral Hospital of Bukavu (PRHB). Pregnant women visiting the PRHB for antenatal care were asked whether they were interested in participation, whereafter eligible women were individually informed about the study details. Women were considered eligible when they (i) were between 16 and 20 weeks pregnant, (ii) accepted to be followed by a referral hospital team, (iii) were willing to deliver at PRHB, and (iv) agreed to be contacted by phone or other means. Women could not be included in the study (i) if they planned to move out of the study area during their pregnancy, (ii) in case of genital bleeding, (iii) in case of twin pregnancies or a visible malformation of the fetus at ultrasound examination, or (iv) in case they used antibiotics during the two weeks before recruitment. The research described here only reports data on the VMB and clinical correlates at V1, and pregnancy outcomes (such as PTB and LBW).

### Routine antenatal care and delivery procedures

At V1, a questionnaire on sociodemographics, reproductive health history, sexual activity, vaginal practices and vaginal complaints was completed by each participant. A general physical examination, including anthropometric measurements, and a gynecological examination, including a speculum examination with a sterile non-moistened speculum, were performed. The vaginal mucosa and cervix were inspected for the presence of sores and tumors, and a diagnosis of vaginal infection was made according to the syndromic-based protocol for the management of pregnancy of the Ministry of Public Health of DRC (Ministère de la Santé RDdC, 2006), which is based on previous WHO recommendations (World Health, 2007). When a vaginal infection was diagnosed, women were treated empirically with a combination (in one vaginal ovule) of clotrimazole (200 mg) (against candidiasis) and clindamycin (100 mg) (against BV) once a day for six days, in accordance with the local protocol. In case a woman was allergic to clindamycin, this was replaced by metronidazole (in a vaginal ovule). During the gynecological examination, the vaginal pH was also determined by means of indicator pH papers (Hilo Indicator^®^ pH paper, Sigma Aldrich). In addition, an ultrasound examination was performed to determine the viability of the fetus and to measure cervical length. Furthermore, five mL of total blood was collected in a VacuTube^®^ (Becton Dickinson) red tube without anticoagulant for HIV, malaria and hemoglobin testing. Next, midstream urine was collected in a sterile container to investigate the presence of nitrite and white blood cells (indicative for urinary tract infections or bacteriuria) using dipsticks (Multistix dipsticks^®^, Siemens). Lastly, three vaginal swabs were taken from the midportion of the lateral vaginal wall, and a cervicovaginal lavage (CVL) sample was collected by rinsing the cervical mucosa with 5 mL of sterile physiological water and collecting as much lavage as possible into a VacuTube^®^. These CVL samples were stored at -20°C for shipment on dry ice to the Laboratory Bacteriology Research (LBR) (Ghent University, Ghent, Belgium) respecting the cold chain. All participants followed routine antenatal care and they received a single dose of mebendazole (500 mg) against intestinal worm infections and a single dose of sulfadiazine-pyrimethamine (500 mg) against malaria. At delivery, the labor was monitored, and delivery features and pregnancy outcomes were collected by nurses and the senior assistant.

### Study specific laboratory procedures

#### Microscopic examinations

At the PRHB, a wet mount slide was prepared within 20 minutes after collection of a vaginal swab. Saline (0.5 mL) was added to the swab and one droplet of this was put on a glass slide and covered with a cover slip. The presence of *Trichomonas vaginalis*, *Candida*, white blood cells and clue cells was determined with microscopy.

A second vaginal swab was rolled on a glass slide and fixated by briefly passing the back of this slide through a flame. Subsequently, these smears were stored and shipped to the LBR, where they were Gram-stained at the Department of Laboratory Medicine (Ghent University Hospital, Ghent, Belgium) using an automated PolyStainer (IUL). These Gram-stained slides were used to diagnose BV according to Nugent as described previously (Mulinganya et al., 2021).

#### DNA extraction

A random selection was made of 331 CVLs to be analyzed by qPCR. DNA was extracted from CVLs using the RNeasy PowerMicrobiome Kit (Qiagen) according to manufacturer’s instructions, with minor modifications (i.e. DNase treatment was omitted). The DNA extracts were then stored at -20°C until use.

#### qPCR assays

Species-specific qPCR assays were used to quantify *Fannyhessea* (*Atopobium*) *vaginae*, *G. leopoldii*, *G. piotii*, *G. swidsinskii*, *G. vaginalis*, *L. crispatus* and *L. iners*. The species-specific qPCR assays for *G. leopoldii*, *G. piotii*, *G. swidsinskii* and *G. vaginalis* were designed and validated in-house (Latka et al., 2022). All reactions were carried out in a total volume of 10 µL, containing 1X LightCycler 480 SYBR Green I master mix (Roche), forward and reverse primer (listed in **Table 1**) and 2 µL of DNA extract, DNA standard (positive control and calibration) or molecular water (negative control). DNA was extracted from cultured *F. vaginae* (CCUG 38953^T^), *G. leopoldii* (UGent 09.48), *G. piotii* (UGent 18.01^T^), *G. swidsinskii* (GS10234), *G. vaginalis* (GvB LMG7832^T^), *L. crispatus* (LMG 9479^T^) and *L. iners* (FB123-CNA-4) using the High Pure PCR Template Preparation Kit (Roche) as previously described (De Keyser, 2020) and used to make a tenfold standard dilution series to generate qPCR standard curves.

**Table 1:** Primers used in this study.

Species	Primer	Sequence (5’-3’)	Final concentration (µM)	Target gene	Reference
*Fannyhessea vaginae*	AV_F	CCCTATCCGCTCCTGATACC	0.7	16S rRNA	(Menard et al., 2008)
	AV_R	CCAAATATCTGCGCATTTCA	0.7		(Menard et al., 2008)
*Gardnerella leopoldii*	GldnaG_F	GATACTGCACTGTATCGA	0.5	dnaG	(Latka et al., 2022)
	GldnaG_R	CAGTATCAATACCAGCC	0.5		(Latka et al., 2022)
*Gardnerella* *piotii*	GpdnaG_F	AGCTGCTTACGATTATAGT	0.5	dnaG	(Latka et al., 2022)
	GpdnaG_R	TTACTCATTCTAAGCTTAATAG	0.5		(Latka et al., 2022)
*Gardnerella swidsinskii*	GsdnaG_F	ATTTAGTTAGATATTTGGCAA	0.5	dnaG	(Latka et al., 2022)
	GsdnaG_R	ATAGTCATATATTCCGCGC	0.5		(Latka et al., 2022)
*Gardnerella vaginalis*	GvdnaG_F	TATTATAACTAAAGCTGCTG	0.5	dnaG	(Latka et al., 2022)
	GvdnaG_R	TCGCCACTATAGTCG	0.5		(Latka et al., 2022)
*Lactobacillus crispatus*	Lcris_F	AGCGAGCGGAACTAACAGATTTAC	0.1	16S rRNA	(Byun et al., 2004)
	Lcris_R	AGCTGATCATGCGATCTGCTT	0.1		(Byun et al., 2004)
*Lactobacillus iners*	Liners_F	GTCTGCCTTGAAGATCGG	0.2	16S rRNA	(De Backer et al., 2007)
	Liners_R	ACAGTTGATAGGCATCATC	0.2		(De Backer et al., 2007)

Reactions were carried out on a LightCycler 480 (Roche). qPCRs were performed for *F. vaginae* by pre-incubation for 10 min at 95°C, followed by 40 cycles of 15 s at 95°C, 20 s at 62°C and 40 s at 72°C, for *Gardnerella* species by pre-incubation 5 min at 95°C, followed by 40 cycles of 15 s at 95°C, 30 s at 56°C and 30 s at 72°C, for *L. crispatus* by pre-incubation for 10 min at 95°C, followed by 40 cycles of 15 s at 95°C, 30 s at 60°C and 30 s at 72°C, and for *L. iners* by pre-incubation for 10 min at 95°C, followed by 40 cycles of 10s at 95°C, 20 s at 50°C and 4 s at 72°C.

High resolution melting curves were generated by first melting all amplified dsDNA at 95°C for 5 s, followed by renaturating the DNA for 30 s at 50°C (*F. vaginae*), 1 min at 55°C (*Gardnerella* species), or 1 min at 60°C (*L. crispatus* and *L. iners*), whereafter the temperature was increased to 97°C at a ramp rate of 0.02°C/s. Specific target amplification was defined based on a melting temperature within a range of 1°C of the mean of the standard dilution series of the respective assays. All raw data was analyzed with the standard LightCycler 480 Software Version 1.5 (Roche). All bacterial concentrations were expressed in genome equivalents/mL (GE/mL).

### Data analysis

First, the presence of each bacterial species as assessed by qPCR was defined as a binary variable and was used to calculate prevalences and univariate associations. The prevalence of each bacterial species was documented together with the corresponding 95% confidence interval (CI), which was calculated using the Wilson method. The median and range of (log-transformed) concentrations of the different species among positive women were documented in boxplots. Next, the Pearson correlation coefficients between the different bacterial concentrations were calculated with the R programming language to document the correlations between the species and were illustrated in a correlation matrix created with heatmapper.ca (http://www.heatmapper.ca/pairwise/). The Pearson correlation coefficients were defined as negligible (0.00-0.10), weak (0.10-0.39), moderate (0.40-0.69), strong (0.70-0.89), or very strong (0.90-1.00) (Schober et al., 2018). To identify the best molecular markers for BV, receiver-operator characteristic (ROC) curves were created based on the log-transformed bacterial concentrations and the categorization of BV based on Nugent (healthy and intermediate scores seen as negative for BV) using the R package pROC (Robin et al., 2011). A logistic regression model was made to investigate the best combination of markers.

The log-transformed bacterial concentrations were used as continuous variables to create VMB profiles and were visualized in a heatmap using the R function pheatmap() (Kolde, 2019). On this heatmap, clusters of VMB profiles were visually defined after hierarchical clustering. Species were also hierarchically clustered on the heatmap. We defined clusters by cutting the tree at a height (indicating dissimilarity) of 110 using the function cutree(). This height was chosen because it yielded two expected clusters of healthy VMB (one dominated by *L. crispatus* and one dominated by *L. iners*).

Subsequently, univariate associations between the bacterial species and clusters on the one hand, and clinical signs and symptoms and pregnancy outcomes on the other hand were determined. For this, the presence of each bacterial species/cluster was defined as the independent variable in each univariate analysis, and clinical signs and symptoms of mother and neonate, as well as pregnancy outcomes, were defined as dependent variables. PTB was defined as birth before 37 completed weeks of gestation and low birthweight as a birthweight below 2500 g. Fisher exact tests were performed to determine which of these associations were significant (p-value <0.05) and odds ratios (OR) were reported for each binary variable. For the parameters that showed a significant p-value when comparing clusters, specific T-tests were performed to compare all clusters against a reference cluster. Here, cluster 6, dominated by *L. crispatus* and mainly containing women with a healthy VMB according to Nugent, was selected as reference cluster for these one-on-one comparative analyses. All statistical analyses described above were performed with R Studio (version 2024.09.1 + 394) or GraphPad Prism [version 10.0.3 (217)].

## Results

### Participant flow and sociodemographics

**Figure 1:**
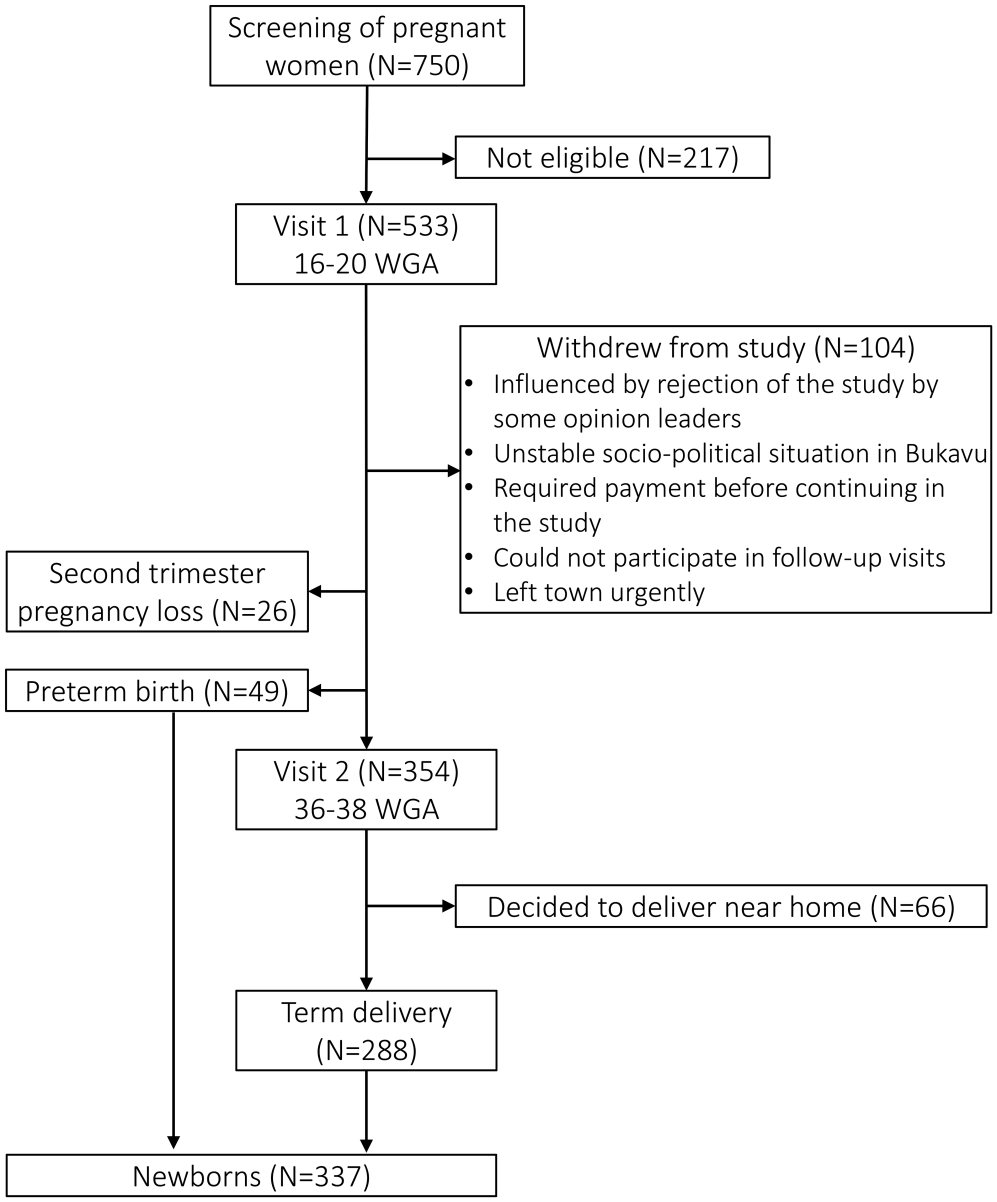
Flowchart of the study. CVL, cervicovaginal lavage; WGA, weeks of gestational age.

The flowchart depicted in **Figure 1** describes the number of pregnant women and neonates withheld at each visit. A total of 750 women were screened, of which 533 were found eligible and included in the study (at V1). Roughly one fifth of these women withdrew from the study before V2, mostly due to rejection of the study by some opinion leaders (who wrongly believed participants were given a substantial imbursement) and the instable socio-political situation in Bukavu during the study period. Of the 533 women included, a second trimester pregnancy loss happened in 26 women (4.9%), and 49 neonates (9.2%) were born preterm. Of the 354 women who completed V2, 66 (18.6%) withdrew from the study because they decided to not deliver at PRHB. The 288 other neonates (81.4%) were all born at term at PRHB.

The complete AVEONS study population has previously been described in detail (Mulinganya et al., 2024). The demographics are summarized in **Table 2**. There were no statistically significant differences in terms of demographics and Nugent score at V1 in the 331 participants whose CVLs were selected for qPCR analysis compared to the overall study population (data not shown). Among these 331 women, 172 gave birth at term and 30 PTBs were observed.

**Table 2:** Sociodemographic characteristics and pregnancy outcomes of the study population stratified by vaginal microbiota categorization.

Characteristics of pregnant women	Number 525 (%)	Vaginal microbiota		
		Healthy microbiome 285 (54.3)	Intermediate microbiome 102 (19.4)	Bacterial vaginosis 138 (26.3)
Age at recruitment				
<20 years	26 (5.0)	13 (4.6)	5 (4.9)	8 (5.8)
20-24 years	113 (21.5)	65 (22.8)	17 (16.7)	31 (22.5)
25-29 years	172 (33)	94 (33.0)	36 (35.3)	43 (31.2)
30-34 years	135 (25.7)	72 (25.3)	30 (29.4)	33 (23.9)
≥35 years	78 (14.9)	41 (14.4)	14 (13.7)	23 (16.7)
Tribe				
Shi	374 (71.2)	203 (71.2)	83 (81.4)	88 (63.8)
Rega	57 (10.9)	32 (11.2)	8 (7.8)	17 (12.3)
Other tribes^1^	94 (17.9)	50 (17.5)	11 (10.8)	33 (23.9)
Religion^2^				
Christians	492 (93.7)	266 (93.3)	94 (92.2)	132 (95.7)
Not Christian	33 (6.3)	19 (6.7)	8 (7.8)	6 (4.4)
Educational level^3^				
Primary	62 (11.8)	35 (12.3)	11 (10.8)	16 (11.6)
Secondary	273 (52.0)	151 (53.0)	54 (52.9)	68 (49.3)
Higher	190 (36.2)	99 (34.7)	37 (36.3)	54 (39.1)
Quality of life^4^				
Poor	381 (72.6)	209 (73.3)	74 (72.6)	98 (71.0)
Not poor	144 (27.4)	76 (26.7)	28 (27.5)	40 (29.0)
Employment status				
Employed or self-employed	89 (17.0)	57 (20.0)	17 (16.7)	15 (10.9)
Unemployed	436 (83.1)	228 (80.0)	85 (83.3)	122 (89.1)
Marital status				
Married	501 (95.4)	273 (95.8)	100 (98.0)	128 (92.8)
Unmarried	24 (4.6)	12 (4.2)	2 (2.0)	10 (7.3)
Clinical status^5^ at first visit				
Symptomatic	254 (48.4)	126 (44.2)	55 (53.9)	73 (52.9)
Asymptomatic	271 (51.6)	159 (55.8)	47 (46.1)	65 (47.1)

^1^Tembo, Fuliru, Hunde, Nyanga, Hutu, Nande, Vira, Bembe, each with a proportion < 2.5%; ^2^Christian represents Catholics, Protestants, Anglicans, Kimbanguistes and Christian Revival Church; not Christian represents Muslims, Animists, and atheists; ^3^Participants who not yet ended their level were included in that level; ^4^Taking into account local parameters, poverty was calculated considering the type of the floor, water source, electricity and commodities in the house. The total score ranged from 4 to 17, a score < 10 was considered under the threshold of poverty, a score ≥10 was considered above the threshold of poverty. We did not include income in the score calculation because it is very unstable and depends mainly on the informal sector, ^5^Participants were symptomatic if they presented with abnormal vaginal discharge, vaginal itching, burning vaginal sensation after sexual intercourse and/or foul smell from vagina.

### Distribution and concentration of the bacterial species

**Figure 2:**
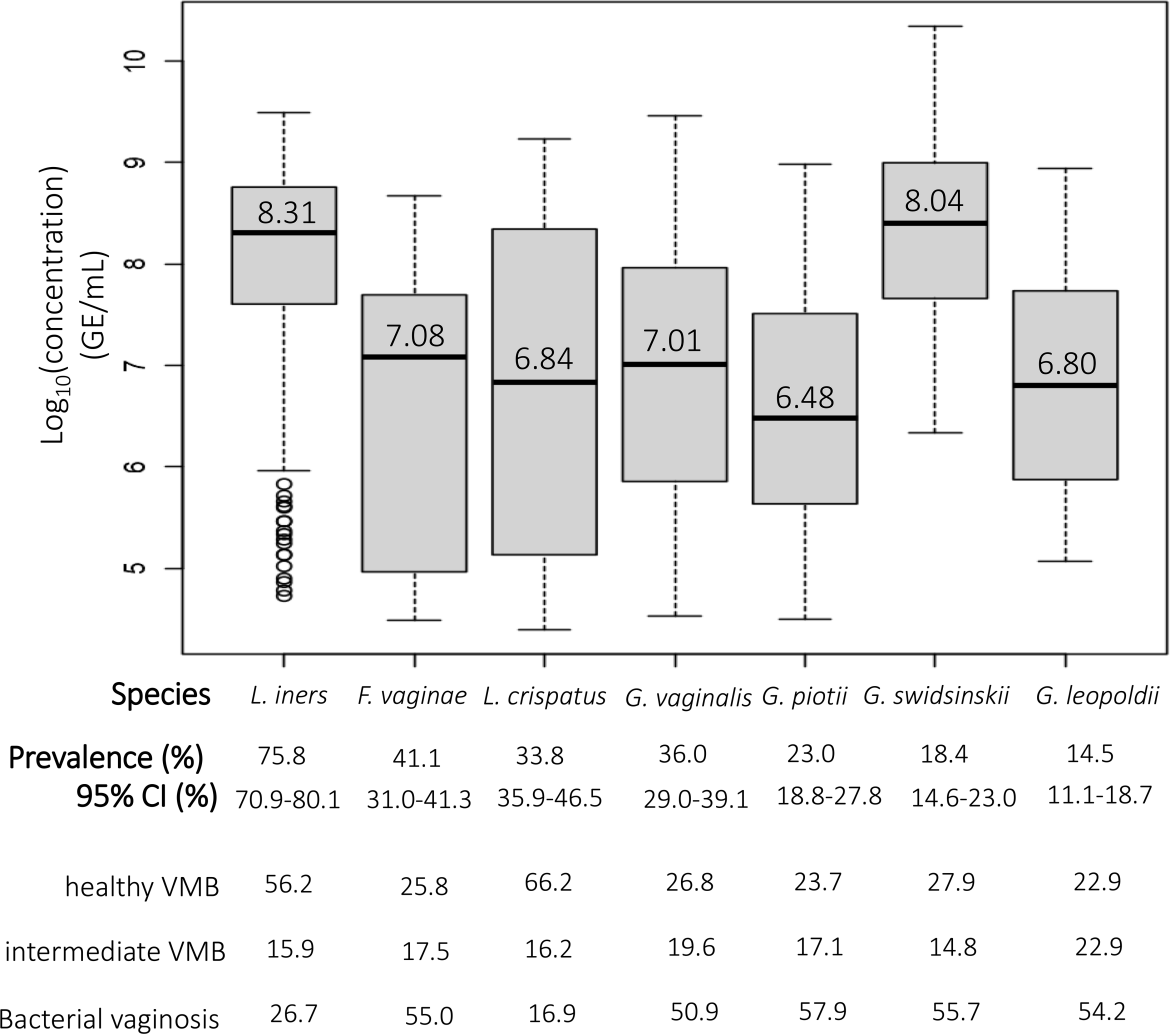
The prevalence and median concentrations of the different species. The black horizontal line in each boxplot represents the median concentration of each species among women positive for that species, which is also shown above this line; the bottom and top edge of the grey boxes represent the first (25% of the data is below this value) and third quartile (75% of the data is below this value), respectively; bottom and top end of the dotted line represent minimum and maximum, respectively; open circles represent outliers.

The prevalence, overall and across VMB categories, and the median and range of the (log_10_-transformed) concentrations of each species among positive women are presented in **Figure 2**. Overall, *L. iners* was the most prevalent species (75.8%) and had the highest median concentration (8.31 log_10_ GE/ml). We found that 36.0% of women were positive for *G. vaginalis*, making it the most common *Gardnerella* species in this population. *G. leopoldii* was the least prevalent *Gardnerella* species, with a prevalence of 14.5%. *G. swidsinskii* had the highest median concentration among *Gardnerella* species (8.04 log_10_ GE/ml) while *G. piotii* had the lowest median concentration (6.48 log_10_ GE/ml).

In total, 157 women (47.4%) were not colonized with *Gardnerella* species, 82 women (24.8%) were colonized with only a single *Gardnerella* species, 62 women (18.7%) were colonized with two *Gardnerella* species, 29 women (8.8%) were colonized with 3 *Gardnerella* species and one woman (0.3%) was colonized with all four *Gardnerella* species. Overall, *Gardnerella* and *F. vaginae* occurred mostly among women with BV, more specifically 54.2% of all *G. leopoldii* was found among women with BV, and this was 57.9% for *G. piotii*, 55.7% for *G. swidsinskii*, 50.9% for *G. vaginalis* and 55.0% for *F. vaginae*. In contrast, *L. crispatus* and *L. iners* were most abundant among women with a healthy VMB (66.2% and 56.2% of *L. crispatus* and *L. iners*, respectively).

### Correlations between the different species

**Figure 3:**
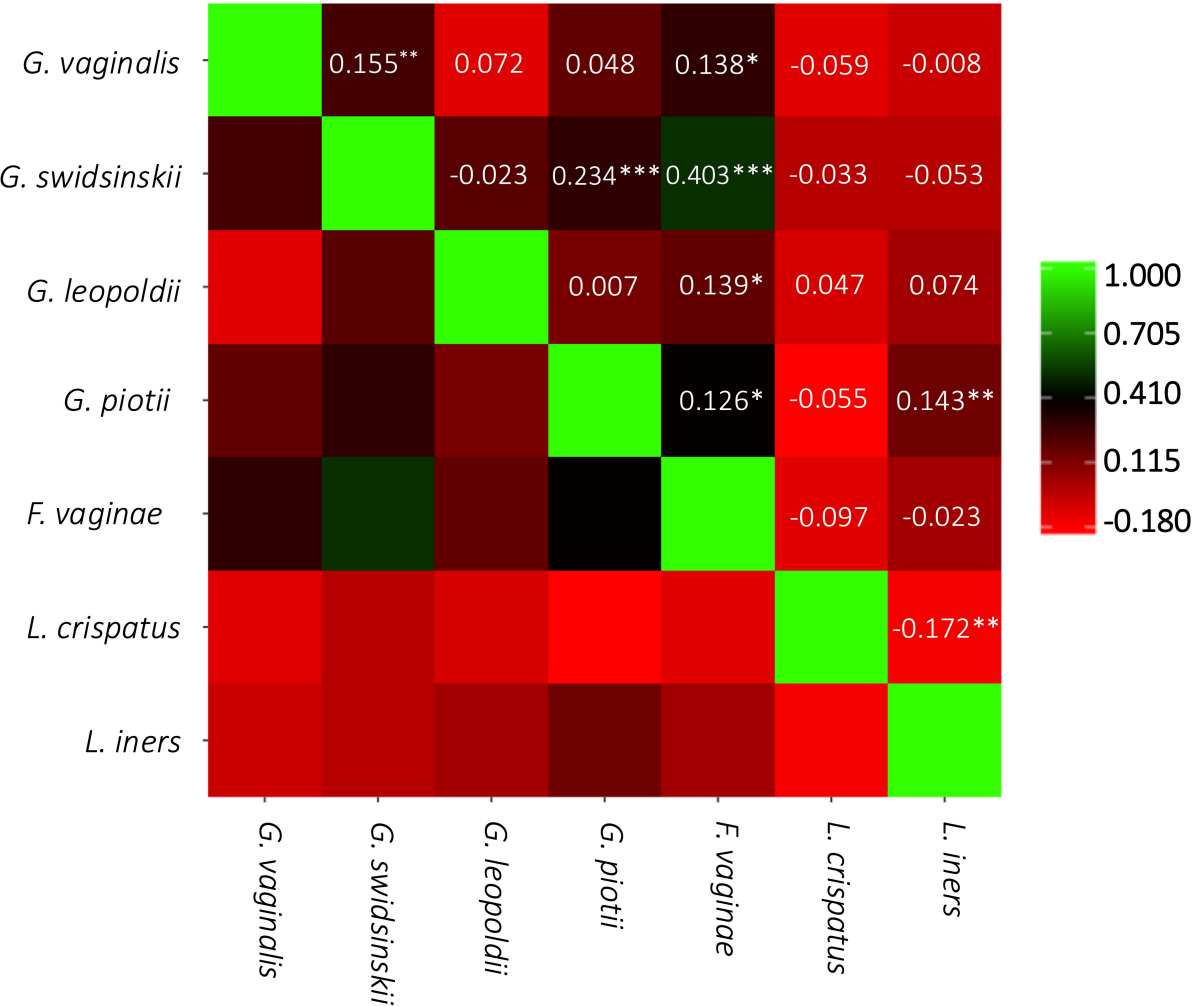
Pairwise correlation matrix. Pearson correlation coefficients are represented by the color scale and are shown in the squares. ***p<0.001; **p<0.01; *p<0.05.

**Figure 3** presents the Pearson correlation coefficients between the concentrations of the different species. The strongest correlation was seen between *G. swidsinskii* and *F. vaginae* (r = 0.403, moderate correlation). There was a significantly weak negative correlation between *L. iners* and *L. crispatus* (r = -0.172), and a significantly weak positive correlation between *L. iners* and *G. piotii* (r = 0.143). Furthermore, a significantly weak positive correlation was seen between *G. swidsinskii* and *G. vaginalis* (r = 0.155), *G. swidsinskii* and *G. piotii* (r = 0.234), *F. vaginae* and *G. leopoldii* (r = 0.139), and *F. vaginae* and *G. piotii* (r = 0.126).

### ROC analysis and logistic regression model

**Figure 4:**
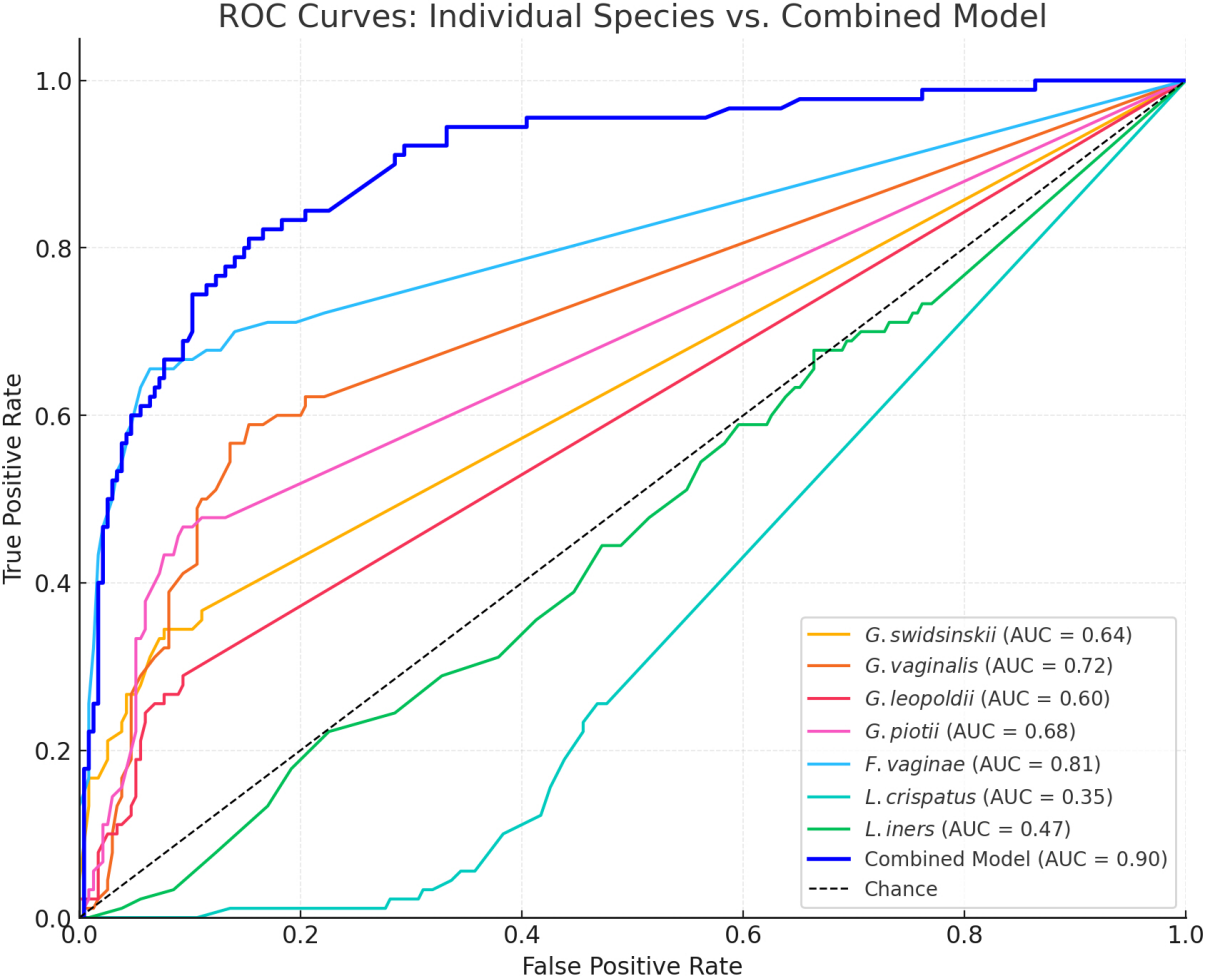
Receiver operator characteristics (ROC) curves for all investigated species and the multiple logistic regression model. AUC, area under the curve. The dashed diagonal line represents random performance (AUC = 0.50).

In **Figure 4** the ROC curves and in **Table 3** the area under the curve (AUC), the Youden’s index, sensitivity and specificity are shown for each species and the multiple logistic regression model. Off all investigated species, *F. vaginae* had the largest area under the curve (0.81), followed by *G. vaginalis* (0.72), *G. piotii* (0.68), *G. swidsinskii* (0.64) and *G. leopoldii* (0.60) The logistic regression model gave the following combined result as optimal to diagnose BV: -1.46 + (0.14 × *G. swidsinskii*) + (0.10 × *G. vaginalis*) + (0.15 × *G. leopoldii*) + (0.15 × *G. piotii*) + (0.29 × *F. vaginae*) - (0.26 × *L. crispatus*) - (0.13 × *L. iners*).

**Table 3:** Diagnostic performance of each investigated species and the multiple logistic regression model for bacterial vaginosis.

Species/Model	AUC	Youden Index	Threshold	Sensitivity (%)	Specificity (%)
*F. vaginae*	0.81	0.59	6.6	65.6	93.6
*G. leopoldii*	0.60	0.20	5.1	28.9	90.6
*G. piotii*	0.68	0.37	5.0	46.7	90.6
*G. swidsinskii*	0.64	0.27	7.3	34.4	92.3
*G. vaginalis*	0.72	0.44	5.5	58.9	84.7
*L. crispatus*	0.35	0.00	10.2	0.0	1.0
*L. iners*	0.47	0.01	6.7	67.8	33.6
Combined model	0.90	0.66	0.51	81.1	84.7

AUC, area under the curve. Threshold values are in logarithmic scale.

### Characterization of clusters based on vaginal microbiome profiles

**Figure 5:**
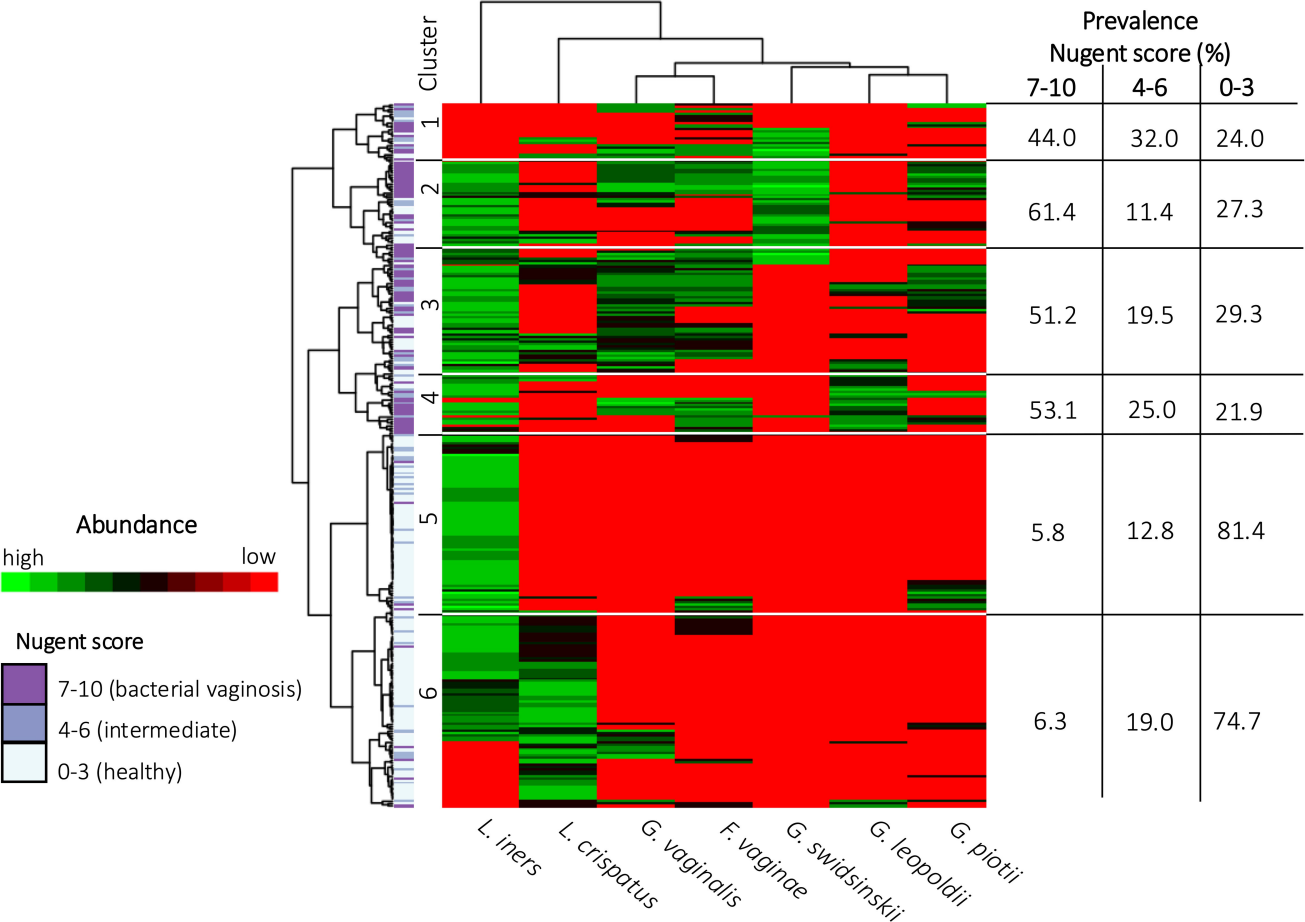
Heatmap of the vaginal microbiome. Each row depicts a woman’s vaginal microbiome profile and each column represents a bacterial species. The hierarchical clustering of vaginal microbiome profiles and bacterial species is based on log-transformed concentrations of bacterial species. The color scale ranges from red, indicating the absence of bacterial species, to green, indicating a high concentration of bacterial species. The categorization of the vaginal microbiome in healthy, intermediate or BV is shown on the left.

In **Figure 5** a heatmap with hierarchical clustering of VMB profiles and bacterial species, annotated by Nugent score categorization, is shown. Six different clusters (1 to 6) were defined after cutting the dendrogram of these profiles at a height of 110. Four of these clusters mainly contained women with Nugent BV (cluster 1-4), while the other clusters contained mainly women with a healthy VMB according to Nugent (cluster 5-6). Each cluster can be characterized by a distinct distribution pattern of species. The prevalence of the species among the different clusters is listed in **Table 4**. Cluster 1 was the only cluster not containing *L. iners*. Also in cluster 1, almost no *G. leopoldii* was present. In cluster 2, also almost no *G. leopoldii* was present, while *G. swidsinskii* and *L. iners* were found in all women of this cluster. Cluster 3 was characterized by 100% abundance of *G. vaginalis*, and an absence of *G. swidsinskii*, while *G. leopoldii* was found among approximately one fifth of the women in this cluster. In cluster 4, *G. leopoldii* was 100% abundant, while *G. swidsinskii* was hardly present, and *G. piotii* and *L. crispatus* were found in one quarter of the women in this cluster. In cluster 5, *L. iners* was 100% abundant, with hardly any *L. crispatus*, and *G. piotii* as the only *Gardnerella* species. In cluster 6, both *L. iners* and *L. crispatus* were highly abundant, while (almost) no *G. swidsinskii*, *G. leopoldii* and *G. piotii* were found among this cluster.

**Table 4:** Prevalence of species in the clusters. N, number of participants.

Cluster (N)	*Lactobacillus iners* (%)	*Lactobacillus crispatus* (%)	*Gardnerella vaginalis* (%)	*Fannyhessea vaginae* (%)	*Gardnerella swidsinskii* (%)	*Gardnerella leopoldii* (%)	*Gardnerella piotii* (%)
1 (26)	0.0	26.9	46.2	65.4	57.7	3.8	23.1
2 (45)	100.0	28.9	60.0	60.0	100.0	4.4	48.9
3 (42)	100.0	47.6	100.0	83.3	0.0	21.4	52.4
4 (32)	87.5	25.0	46.9	46.9	3.1	100.0	25.0
5 (87)	100.0	1.3	0.0	13.9	0.0	0.0	16.5
6 (79)	66.7	100.0	18.4	17.2	0.0	4.6	5.7

### Univariate associations of the different species and clusters with laboratory findings and clinical signs and symptoms

The statistically significant univariate associations of the presence of the different species with laboratory findings and clinical signs and symptoms are summarized in **Table 5**. Further results of these univariate analyses (which were not statistically significant) for the different species are shown in **Supplementary Information 1** (SI1) to SI7. The results of the univariate analysis across all clusters are shown in SI8.

**Table 5:** Summary of univariate associations between bacterial species and clinical signs and symptoms.

	Bacterial species present	Bacterial species absent	p-value	Odds ratio (95% CI)
*Gardnerella leopoldii* (48 positive, 283 negative)				
Vaginal microbiome characterization				
Healthy VMB, n (%) (N=176)	11 (22.92)	165 (59.35)	<0.001	REF
Intermediate VMB, n (%) (N=59)	11 (22.92)	48 (17.27)		5.95 (2.66-14.17)
Bacterial vaginosis, n (%) (N=91)	26 (54.17)	65 (23.38)		3.42 (1.26-9.29)
Mean Nugent score	5.69	2.98	0.001	NA
*Gardnerella piotii* (76 positive, 255 negative)				
Dysuria, n (%) (N=86)	29 (39.19)	57 (22.80)	0.007	2.18 (1.20-3.92)
Anemia, n (%) (N=24)	10 (13.51)	14 (5.49)	0.038	2.68 (1.01-6.84)
Nitrite urine dipstick, n (%) (N=12)	8 (10.67)	4 (1.57)	0.001	7.43 (1.92-34.77)
Vaginal microbiome characterization				
Healthy VMB, n (%) (N=176)	18 (24.00)	158 (62.95)	<0.001	REF
Intermediate VMB, n (%) (N=59)	13 (17.33)	46 (18.33)		8.14 (4.16-16.49)
Bacterial vaginosis, n (%) (N=91)	44 (58.67)	47 (18.73)		2.47 (1.03-5.80)
White blood cells urine dipstick				
≥ 25, n (%) (N=19)	3 (4.00)	16 (6.27)	0.019	REF
≥ 50, n (%) (N=45)	12 (16.00)	33 (12.94)		0.52 (0.08-2.32)
≥ 75, n (%) (N=70)	25 (33.33)	45 (17.65)		0.34 (0.06-1.36)
Negative, n (%) (N=196)	35 (46.67)	161 (63.14)		0.86 (0.15-3.26)
Mean Nugent score	5.92	2.62	<0.001	NA
*Gardnerella swidsinskii* (61 positive, 270 negative)				
Vaginal microbiome characterization				
Healthy VMB, n (%) (N=176)	17 (28.33)	159 (59.77)	<0.001	REF
Intermediate VMB, n (%) (N=59)	9 (15.00)	50 (18.80)		5.54 (2.77-11.45)
Bacterial vaginosis, n (%) (N=91)	34 (56.67)	57 (21.43)		1.68 (0.62-4.28)
Mean Nugent score	5.57	2.89	<0.001	NA
*Gardnerella vaginalis* (112 positive, 219 negative)				
Positive whiff test, n (%) (N=31)	18 (16.07)	13 (6.02)	0.005	2.98 (1.32-6.91)
Vaginal microbiome characterization				
Healthy VMB, n (%) (N=176)	30 (27.52)	146 (67.28)	<0.001	REF
Intermediate VMB, n (%) (N=59)	22 (20.18)	37 (17.05)		8.08 (4.40-15.17)
Bacterial vaginosis, n (%) (N=91)	57 (52.29)	34 (15.67)		2.88 (1.41-5.85)
Mean Nugent score	5.47	2.33	<0.001	NA
*Fannyhessea vaginae* (120 positive, 211 negative)				
Positive whiff test, n (%) (N=31)	20 (16.95)	11 (5.24)	0.007	3.68 (1.61-8.85)
Vaginal microbiome characterization				
Healthy VMB, n (%) (N=176)	31 (26.27)	145 (69.71)	<0.001	REF
Intermediate VMB, n (%) (N=59)	21 (17.80)	38 (18.27)		12.19 (6.50-23.61)
Bacterial vaginosis, n (%) (N=91)	66 (55.93)	25 (12.02)		2.57 (1.26-5.23)
White blood cells urine dipstick				
≥ 25, n (%) (N=19)	7 (5.88)	12 (5.69)	0.017	REF
≥ 50, n (%) (N=45)	19 (15.97)	26 (12.32)		0.80 (0.22-2.72)
≥ 75, n (%) (N=70)	35 (29.41)	35 (16.59)		0.59 (0.17-1.84)
Negative, n (%) (N=196)	58 (48.74)	138 (65.40)		1.39 (0.44-4.05)
Mean number of white blood cells on wet mount per field	9.82	8.35	0.045	NA
Mean Nugent score	5.56	2.14	<0.001	NA
Mean vaginal pH	6.07	5.87	0.001	NA
*Lactobacillus crispatus* (136 positive, 195 negative)				
Vaginal microbiome characterization				
Healthy VMB, n (%) (N=176)	90 (66.67)	86 (45.03)	<0.001	REF
Intermediate VMB, n (%) (N=59)	22 (16.30)	37 (19.37)		0.32 (0.18-0.58)
Bacterial vaginosis, n (%) (N=91)	23 (17.04)	68 (35.60)		0.57 (0.29-1.08)
Mean Nugent score	2.43	4.05	<0.001	NA
Mean vaginal pH	5.88	5.98	0.043	NA
*Lactobacillus iners* (251 positive, 76 negative)				
Maternal fever, n (%) (N=37)	34 (13.77)	3 (4.11)	0.022	3.71 (1.11-19.47)
*Candida* on wet mount, n (%) (N=91)	77 (30.80)	14 (18.67)	0.041	1.94 (1.00-3.98)

CI, confidence interval.

NA, not applicable.

All species except for *L. iners* were significantly associated with the Nugent score and the corresponding categorization of the VMB. Furthermore, *G. piotii* was positively associated with clinical symptoms and markers of urinary tract infections, i.e. dysuria (OR: 2.18; 95% CI: 1.20-3.92), nitrite levels measured with urine dipstick (OR: 7.43; 95% CI: 1.92-34.77) and white blood cell levels measured with urine dipstick. Also, women colonized with *G. piotii* had higher odds for anemia compared to women not colonized with *G. piotii* (OR: 2.68; 95% CI: 1.01-6.48). *G. vaginalis* showed a positive association with the Whiff test (OR: 2.98; 95% CI: 1.32-6.91). Women colonized with *F. vaginae* also showed increased odds for a positive Whiff test compared to women not colonized with *F. vaginae* (OR: 3.68; 95% CI: 1.61-8.85). Furthermore, *F. vaginae* was significantly associated with an increase in vaginal pH and white bloods cell levels determined on wet mount as well as with urine dipstick. *L. crispatus*, on the other hand, showed a significant association with a decrease in vaginal pH. For *L. iners* a significant positive association was found with maternal fever at V1 (OR: 3.71; 95% CI: 1.11-19.47) and *Candida* on wet mount (OR: 1.94; 95% CI: 1.00-3.98).

After one-on-one comparison with reference cluster 6, significant differences were found for Whiff test with cluster 2, with cluster 3 and with cluster 4. Cluster 6 and cluster 2 differed significantly with regard to *Candida* on wet mount. For Nugent score and VMB-categorization according to Nugent, cluster 1 to cluster 4 differed significantly from cluster 6. For fever the significant difference was between clusters other than reference cluster 6.

### Univariate associations of the different species and clusters with pregnancy outcomes

The associations between the different species and pregnancy outcomes are shown in SI1-7. A significant positive association was found between LBW and two species, i.e. *G. vaginalis* (OR: inf; 95% CI: 3.03-inf, based on all seven women with a LBW baby being colonized with *G. vaginalis*) and *F. vaginae* (OR: 9.50; 95% CI: 1.12-444.16, based on six of the seven women with a LBW baby being positive for *F. vaginae*) (SI4 and SI5). Furthermore, women colonized with *L. iners* showed an almost four times higher odds for delivering a baby preterm compared to women not colonized with *L. iners* (OR: 3.73; 95% CI: 1.07-20.08) (SI7). However, also 70.6% of women with term birth were colonized by *L. iners*, although the mean *L. iners* concentration was twice as high in women with PTB compared to women who delivered at term (p<0.05) (data not shown). For the different clusters, no significant associations were found with any of the pregnancy outcomes.

## Discussion

### Prevalence and cooccurrence

In this study we investigated the distribution and clinical correlates of four *Gardnerella* species, *L. crispatus*, *L. iners* and *F. vaginae* related to women’s reproductive health. To our best knowledge, the different *Gardnerella* species have not yet been studied in African women. In our study population of Congolese pregnant women, *G. vaginalis* was the most prevalent *Gardnerella* species (33.8%), followed by *G. piotii* (23.0%), *G. swidsinskii* (18.4%), and *G. leopoldii* (14.5%). This is in line with a study among Dutch women where *G. leopoldii* was also the least prevalent species (22.2%) (Schuster et al., 2024). Also, if colonized with *Gardnerella*, the proportion of women harboring one *Gardnerella* species was approximately equal to the proportion of women carrying more than one *Gardnerella* species. This is in line with a study by Berman et al., who, based on sequences derived from vaginal swab samples from three demographically distinct cohorts of pregnant women, showed that metagenomes in which *Gardnerella* was present often contained more than one species (mean 3.92) (Berman et al., 2024), and with other previous work that has shown the majority of women are being colonized by more than one *Gardnerella* species (Munch et al., 2024; Schuster et al., 2024). Also noteworthy here is that *G. swidsinskii* had a median concentration around ten times higher compared to the other *Gardnerella* species.

In 2019, Hill and coworkers investigated the distribution of *Gardnerella* species among *Gardnerella*-positive non-pregnant Canadian women based on cpn60 deep sequencing (Hill and Albert, 2019). They reported *G. vaginalis* to be the most prevalent *Gardnerella* species (68.4%), followed by *G. swidsinskii* (49.2%), *G. leopoldii* (26.2%) and *G. piotii* (25.2%). These prevalences are considerably higher compared to ours, likely because only *Gardnerella*-positive women (72.9% of their total study population) were analyzed. In their study, they also reported a statistically significant co-occurrence between *G. swidsinskii* and *G. vaginalis*, and *G. piotii* and genomospecies 3, while *G. swidsinskii* and *G. leopoldii* significantly did not occur together. This is partly in line with findings reported here, since we also observed a positive correlation between *G. swidsinskii* and *G. vaginalis*. We did, however, also find a statistically significant correlation between *G. swidsinskii* and *G. piotii*.

In our study population, *L. iners* was the most prevalent species (75.8%), which is in line with results from numerous previous studies in African populations (Vaneechoutte, 2017a) (Vaneechoutte, 2017b). We found a negative correlation between *L. iners* and *L. crispatus* (r = -0.172), which is also in line with most literature, documenting single species dominance of lactobacilli in the healthy VMB (Vaneechoutte, 2017a). Only very weak correlations were seen between *L. crispatus* and *Gardnerella* species, while a positive correlation did exist between *L. iners* and *G. piotii* (r = 0.143). For *F. vaginae* a co-occurrence was reported with *G. swidsinskii* (r = 0.403), with *G. leopoldii* (r = 0.139) and with *G. piotii* (r = 0.126), which is in line with the general knowledge that *F. vaginae* often occurs together with *Gardnerella* (Bradshaw et al., 2006).

### Diagnostic markers

BV-associated species such as *G. vaginalis*, *F. vaginae*, *Megasphaera* type 1, bacterial vaginosis-associated bacterium-2 (BVAB-2) and/or species associated with health, such as *Lactobacillus* spp., are commonly utilized as qPCR targets in both commercial [e.g. BD MAX™ Vaginal Panel (BD), Xpert Xpress MVP (Cepheid)] and in-house assays for BV detection. However, some of these markers, for example BVAB-2, have been shown to differ in prevalence in women found positive for BV depending on race (Srinivasan et al., 2012).

In our study population, we showed that *F. vaginae* was the best species marker for BV according to ROC analysis, with a sensitivity of 66% and specificity of 94%, using Nugent-BV as reference. This is in line with several previous studies that have shown that *F. vaginae* is a more specific marker for BV compared to the different *Gardnerella* species investigated (De Backer et al., 2007; Marconi et al., 2012; Vaneechoutte, 2017b). When considering combinations of species, our logistic regression model increased the AUC to 0.90, with a sensitivity and specificity of 81% and 85%, respectively.

Other studies using similar approaches of combining species markers to diagnose BV reported higher sensitivities and specificities (Fredricks et al., 2007; Hilbert et al., 2016; Munch et al., 2024). This might partly be due to the fact that we did not consider markers such as *Megasphaera* and BVAB-2, which have been found to have sensitivities and specificities of around 96% and 94%, respectively, compared to Nugent-BV (Fredricks et al., 2007). However, the same study also documented that *Fannyhessea* alone had a sensitivity and specificity of 96% and 85%, respectively, outperforming our combined model (Fredricks et al., 2007). Also in the study of Hilbert and coworkers, *Fannyhessea* alone had a sensitivity and specificity of 87% and 91%, respectively, and the overall model had a sensitivity and specificity of 92% and 95%, respectively (Hilbert et al., 2016). Taken together, above findings might suggest that optimal (combined) molecular BV-markers are population dependent.

The AUCs for the investigated *Gardnerella* species were similar (ranging from 0.60 for *G. leopoldii* to 0.72 for *G. vaginalis*), with *G. vaginalis* having the highest sensitivity but the lowest specificity. *G. vaginalis* was also suggested as the best marker among the different *Gardnerella* species by Munch and colleagues (Munch et al., 2024). Furthermore, all *Gardnerella* species and *F. vaginae* were positively, and *L. crispatus* negatively associated with the Nugent score and Nugent categorization, which confirms their role as key markers of (Nugent) BV also in our study population of women from Bukavu (DRC).

### Univariate species associations

None of the seven investigated species showed a significant association with the symptoms typically associated with BV (i.e. discharge and malodor), or less typically associated with BV (itching and burning). Not stratifying our data for *Candida* carriage here might explain this observation, since this is a known confounder of BV and we previously showed that in *Candida*-negative women BV was significantly associated with malodor, while in *Candida*-positive women BV was not significantly associated to any typical symptom (De Keyser, 2020).

We did find a statistically significant association between *G. piotii* and both symptoms (dysuria) and laboratory markers (nitrite and white blood cells on dipstick) of urinary tract infections (UTIs). *Gardnerella* has previously been identified as a pathogen causing UTIs (Woolfrey et al., 1986) and, in a mouse model, *G. piotii* has been shown to facilitate *Escherichia coli* UTIs (O'Brien et al., 2020). *G. piotii*, in contrast to other *Gardnerella* spp., possesses the gene for extracellular sialidase activity (Kurukulasuriya et al., 2021), which in *Bifidobacterium bifidum* has been shown to enhance mucosal adhesion in *in vitro* experiments (Nishiyama et al., 2017). Hence, this trait could enhance bladder mucosal adhesion of *G. piotii* or other pathogens. Facilitation of adherence by other pathogens might be the more likely explanation, given that *Gardnerella* is thought to be negative for nitrate reduction (Taylor-Robinson, 1984), although this has not been investigated for the different species.

A significant positive association was also seen for *G. piotii* with anemia (hemoglobin levels <12.0 g/dL). Verstraelen and coworkers documented that subclinical iron-deficiency in Belgian pregnant women was an independent predictor for BV (Verstraelen et al., 2005) and it has also been demonstrated that *Gardnerella* can use human hemoglobin as a source of iron (Jarosik et al., 1998). Brabin and coworkers, in contrast, showed that iron-deficient women from Burkina Faso were more likely to have a normal VMB compared to iron-replete women, but also that the prevalence of vaginal discharge was significantly higher among iron-deficient women (Brabin et al., 2017).

Both *G. vaginalis* and *F. vaginae* were found to show a positive association with a positive Whiff test, one of the four Amsel criteria used for the clinical diagnosis of BV (Amsel et al., 1983). In a Whiff test, 10% potassium hydroxide (KOH) is added to vaginal discharge, and a fishy smell (positive Whiff test) is caused by aromatization of aromatic amines by anaerobes associated with BV. *In vitro* and bio-informatic analyses suggest that *Prevotella* but not *Gardnerella* is involved in the production of these amines (Nelson et al., 2015), but this has not yet been studied using various *Gardnerella* species.

### Univariate cluster associations

All *Gardnerella* species were found among the clusters containing mainly Nugent-BV-positive women (i.e. cluster 1 to cluster 4), suggesting that not a single *Gardnerella* species but rather an interplay between different species plays a role in (asymptomatic) BV. This is in line with previous studies showing the majority of women are colonized by more than one *Gardnerella* species (Berman et al., 2024; Munch et al., 2024; Schuster et al., 2024). *G. swidsinskii* and *G. leopoldii* were not and nearly not (4.6%), respectively, observed among clusters with mainly women without Nugent-BV (i.e. cluster 5 and cluster 6), confirming the above described finding that these species are more specific markers for BV than *G. vaginalis*.

We hypothesized that distinct *Gardnerella* species specific clusters within women with Nugent BV existed and were associated to different degrees with clinical signs and symptoms and/or pregnancy outcomes. However, we showed that, although clusters could be defined based on the *Gardnerella* species composition, no signs and symptoms, with the exception of Whiff test, nor pregnancy outcomes were associated with these clusters. This is in line with the fact that we also did not find single *Gardnerella* species to be associated with clinical signs and symptoms and/or pregnancy outcomes (with the exception of *G. vaginalis* being associated with pH, and with LBW, albeit with a very broad 95% CI for the OR).

In cluster 5, which was dominated by *L. iners* and mostly contained women with a healthy Nugent score (74.7%), we saw no statistically significantly lower pH compared to the clusters 1-4, which is somewhat unexpected since this cluster represents mostly healthy women, but is in line with a report stating that the pH is not always lowered in case of *L. iners* predominance (Vaneechoutte, 2017b). Likewise, for cluster 6, which was dominated by *L. crispatus* and also mainly represented healthy women according to Nugent (81.4%), no statistically significant difference in pH was found compared to clusters 1-4.

A previous meta-analysis showed a twofold higher odd for PTB in women with BV (Vandenwyngaerden et al., 2020). In our study population, however, we previously showed that Nugent-BV was not significantly associated with PTB, but with LBW (Mulinganya et al., 2021). Here, using *Gardnerella*-species specific qPCR, offering absolute quantification and a higher taxonomic resolution compared to the Nugent scoring system, we showed that there was no association between different key markers of BV (*Gardnerella*, *F. vaginae*) and a healthy VMB (*L. crispatus*), and PTB. This is in contrast to a study in a cohort of pregnant Dutch women at high risk of recurrent preterm birth, where *G. leopoldii* was in fact associated with spontaneous preterm birth (Schuster et al., 2024).

In contrast, here, we did find that women with PTB had a significantly nearly four times higher odds of *L. iners* compared to women giving birth at term, and we see a higher concentration of *L. iners* among women with preterm birth than among the *L. iners*-carrying women with term birth. These findings are in line with results presented by Petricevic and colleagues, who studied pregnant Austrian women and reported a significantly higher prevalence of *L. iners* among women with PTB (85%) compared to women with term birth (16%) (Petricevic et al., 2014). In another study (among English women with an increased risk of PTB), *L. iners* dominance at 16 weeks of gestation, but not vaginal dysbiosis, was found to be significantly associated with PTB (<34 + 0 weeks) (Kindinger et al., 2017). Fettweis and colleagues, on the other hand, demonstrated that women who delivered at term showed a significant increase in the prevalence of *L. iners* (Fettweis et al., 2019).

### Study limitations

Our study was subject to several limitations. First, approximately one third of study participants dropped out between the first visit and follow up, causing the number of women who delivered at the PRHB hospital to be less than foreseen. Second, women with complaints were treated after the first visit, which could have masked certain correlations. Lastly, we only studied a limited scope of seven key species, excluding other potential marker species such as *Prevotella* spp., *L. gasseri*, *L. jensenii* and *L. vaginalis*.

## Conclusion

*G. vaginalis* was the most prevalent *Gardnerella* species in pregnant women from Bukavu (DRC), and *G. leopoldii* was the least prevalent species. Our results suggest that all *Gardnerella* species are involved in BV, although none were associated with the most clinically important BV symptoms. However, *G. piotii* was associated with markers of urinary tract infection. *F. vaginae* was the best single species diagnostic marker for BV.

## Data Availability

The raw data supporting the conclusions of this article will be made available by the authors, without undue reservation.
