# Supplementary material for: Prevalence and clinical correlates of Gardnerella spp., Fannyhessea vaginae, Lactobacillus crispatus and L. iners in pregnant women in Bukavu, Democratic Republic of the Congo
Source: Front Cell Infect Microbiol. 2025 Jan 17;14:1514884. doi: 10.3389/fcimb.2024.1514884 (PMC11782042; doi:10.3389/fcimb.2024.1514884)
Supplement: Supplementary file 1 [file Table1.docx]

**Supplementary Information 1. Univariate associations between Gardnerella leopoldii and clinical signs and symptoms of mother and baby and pregnancy outcomes.** N, total number of study participants within group; n, number of study participants; OR, odds ratio; CI, confidence interval; NA, not applicable.

| **N=331** | ***Gardnerella leopoldii* (N=48)** | **No *Gardnerella leopoldii* (N=283)** | **P-value** | **Odds ratio (95%CI)** |
| --- | --- | --- | --- | --- |
| Vaginal discharge, n (%) (N=159) | 26 (55.32) | 133 (47.67) | 0.348 | 1.36 (0.70-2.67) |
| Vaginal itching, n (%) (N=136) | 17 (35.42) | 119 (42.50) | 0.429 | 0.74 (0.37-1.46) |
| Dysuria, n (%) (N=86) | 14 (29.79) | 72 (25.99) | 0.595 | 1.21 (0.56-2.48) |
| Burning sensation after sex, n (%) (N=104) | 20 (43.48) | 84 (31.46) | 0.128 | 1.67 (0.83-3.32) |
| Vaginal malodor, n (%) (N=77) | 15 (33.33) | 62 (24.60) | 0.267 | 1.53 (0.72-3.16) |
| Positive whiff test, n (%) (N=31) | 8 (16.67) | 23 (8.21) | 0.103 | 2.23 (0.80-5.61) |
| Anemia, n (%) (N=24) | 5 (10.42) | 19 (6.76) | 0.368 | 1.60 (0.44-4.75) |
| Maternal fever, n (%) (N=37) | 9 (18.75) | 28 (10.14) | 0.089 | 2.04 (0.79-4.87) |
| Uterine contractions, n (%) (N=40) | 5 (11.63) | 35 (14.11) | 0.813 | 0.80 (0.23-2.24) |
| Use of antibiotics 2 weeks  prior to visit, n (%) (N=46) | 7 (14.58) | 39 (13.93) | 0.826 | 1.05 (0.37-2.61) |
| *Trichomonas* on wet mount, n (%) (N=4) | 0 (0.00) | 4 (1.42) | 1.000 | 0.00 (0.00-8.97) |
| *Candida* on wet mount, n (%) (N=91) | 17 (35.42) | 74 (26.33) | 0.222 | 1.53 (0.75-3.05) |
| Infection of baby during  first week of life, n (%) (N=81) | 10 (27.03) | 71 (30.60) | 0.705 | 0.84 (0.34-1.91) |
| Nitrite urine dipstick, n (%) (N=12) | 2 (4.17) | 10 (3.55) | 0.689 | 1.18 (0.12-5.81) |
| State vaginal secretions |  |  |  |  |
| Fine and homogenous, n (%) (N=297) | 42 (87.50) | 255 (90.43) | 0.672 | REF |
| Thick, n (%) (N=16) | 3 (6.25) | 13 (4.61) |  | 1.40 (0.25-5.40) |
| Thick and heterogenous, n (%) (N=17) | 3 (6.25) | 14 (4.96) |  | 1.30 (0.23-4.94) |
| Vulvar state |  |  |  |  |
| Normal, n (%) (N=323) | 47 (100) | 276 (97.87) | 1.000 | REF |
| Erythema, n (%) (N=1) | 0 (0.00) | 1 (0.35) |  | 0.00 (0.00-229.03) |
| Postule, n (%) (N=2) | 0 (0.00) | 2 (0.71) |  | 0.00 (0.00-31.69) |
| Leucorrhoea, n (%) (N=3) | 0 (0.00) | 3 (1.06) |  | 0.00 (0.00-14.52) |
| Vaginal microbiome characterization |  |  |  |  |
| Healthy VMB, n (%) (N=176) | 11 (22.92) | 165 (59.35) | **<0.001** | REF |
| Intermediate VMB, n (%) (N=59) | 11 (22.92) | 48 (17.27) |  | 5.95 (2.66-14.17) |
| Bacterial vaginosis, n (%) (N=91) | 26 (54.17) | 65 (23.38) |  | 3.42 (1.26-9.29) |
| White blood cells urine dipstick |  |  |  |  |
| ≥ 25, n (%) (N=19) | 0 (0.00) | 19 (6.74) | 0.064 | REF |
| ≥ 50, n (%) (N=45) | 8 (16.67) | 37 (13.12) |  | 0.00 (0.00-1.30) |
| ≥ 75, n (%) (N=70) | 15 (31.25) | 55 (19.50) |  | 0.00 (0.00-0.91) |
| Negative, n (%) (N=196) | 25 (52.08) | 171 (60.64) |  | 0.00 (0.00-1.59) |

| **N=331** | ***Gardnerella leopoldii* (N=48)** | **No *Gardnerella leopoldii* (N=283)** | **P-value** | **Odds ratio (95%CI)** |
| --- | --- | --- | --- | --- |
| Mean number of white blood cells on wet mount per field | 9.17 | 8.83 | 0.226 | NA |
| Mean number of epithelial cells on wet mount per field | 27.06 | 26.04 | 0.216 | NA |
| Mean Nugent score | 5.69 | 2.98 | **0.001** | NA |
| Mean vaginal pH | 6.11 | 5.91 | 0.219 | NA |
| Mean length cervix, cm | 38.88 | 38.27 | 0.836 | NA |
| Mean birthweight, g | 3180.45 | 3239.29 | 0.120 | NA |
| Preterm birth, n (%) (N=30) | 4 (12.12) | 26 (15.38) | 0.792 | 0.76 (0.18-2.44) |
| Low birthweight, n (%) (N=7) | 2 (6.45) | 5 (2.92) | 0.293 | 2.28 (0.21-14.74) |
