# Supplementary material for: Prevalence and clinical correlates of Gardnerella spp., Fannyhessea vaginae, Lactobacillus crispatus and L. iners in pregnant women in Bukavu, Democratic Republic of the Congo
Source: Front Cell Infect Microbiol. 2025 Jan 17;14:1514884. doi: 10.3389/fcimb.2024.1514884 (PMC11782042; doi:10.3389/fcimb.2024.1514884)
Supplement: Supplementary file 2 [file Table2.docx]

**Supplementary Information 2. Univariate associations between Gardnerella piotii and clinical signs and symptoms of mother and baby and pregnancy outcomes.** N, total number of study participants within group; n, number of study participants; OR, odds ratio; CI, confidence interval; NA, not applicable.

| **N=331** | ***Gardnerella piotii* (N=76)** | **No *Gardnerella piotii* (N=255)** | **P-value** | **Odds ratio (95%CI)** |
| --- | --- | --- | --- | --- |
| Vaginal discharge, n (%) (N=159) | 39 (52.70) | 120 (47.62) | 0.509 | 1.22 (0.71-2.13) |
| Vaginal itching, n (%) (N=136) | 32 (42.67) | 104 (41.11) | 0.894 | 1.07 (0.61-1.85) |
| Dysuria, n (%) (N=86) | 29 (39.19) | 57 (22.80) | **0.007** | 2.18 (1.20-3.92) |
| Burning sensation after sex, n (%) (N=104) | 26 (36.11) | 78 (32.37) | 0.571 | 1.18 (0.65-2.11) |
| Vaginal malodor, n (%) (N=77) | 19 (28.36) | 58 (25.22) | 0.636 | 1.17 (0.60-2.23) |
| Positive whiff test, n (%) (N=31) | 11 (14.67) | 20 (7.91) | 0.113 | 2.00 (0.82-4.64) |
| Anemia, n (%) (N=24) | 10 (13.51) | 14 (5.49) | **0.038** | 2.68 (1.01-6.84) |
| Maternal fever, n (%) (N=37) | 9 (12.50) | 28 (11.11) | 0.834 | 1.14 (0.45-2.65) |
| Uterine contractions, n (%) (N=40) | 6 (9.09) | 34 (15.11) | 0.308 | 0.56 (0.18-1.45) |
| Use of antibiotics 2 weeks  prior to visit, n (%) (N=46) | 10 (13.33) | 36 (14.23) | 1.000 | 0.93 (0.39-2.04) |
| *Trichomonas* on wet mount, n (%) (N=4) | 1 (1.35) | 3 (1.18) | 1.000 | 1.15 (0.02-14.57) |
| *Candida* on wet mount, n (%) (N=91) | 21 (28.38) | 70 (27.45) | 0.883 | 1.05 (0.56-1.92) |
| Infection of baby during  first week of life, n (%) (N=81) | 20 (32.26) | 61 (29.47) | 0.753 | 1.14 (0.58-2.17) |
| Nitrite urine dipstick, n (%) (N=12) | 8 (10.67) | 4 (1.57) | **0.001** | 7.43 (1.92-34.77) |
| State vaginal secretions |  |  |  |  |
| Fine and homogenous, n (%) (N=297) | 66 (88.00) | 231 (90.59) | 0.446 | REF |
| Thick, n (%) (N=16) | 3 (4.00) | 13 (5.10) |  | 0.81 (0.14-3.06) |
| Thick and heterogenous, n (%) (N=17) | 6 (8.00) | 11 (4.31) |  | 1.90 (0.56-5.87) |
| Vulvar state |  |  |  |  |
| Normal, n (%) (N=323) | 72 (96.00) | 251 (98.82) | 0.027 | REF |
| Erythema, n (%) (N=1) | 0 (0.00) | 1 (0.39) |  | 0.00 (0.00-136.21) |
| Postule, n (%) (N=2) | 0 (0.00) | 2 (0.79) |  | 0.00 (0.00-18.78) |
| Leucorrhoea, n (%) (N=3) | 3 (4.00) | 0 (0.00) |  | Inf (1.40-inf) |
| Vaginal microbiome characterization |  |  |  |  |
| Healthy VMB, n (%) (N=176) | 18 (24.00) | 158 (62.95) | **<0.001** | REF |
| Intermediate VMB, n (%) (N=59) | 13 (17.33) | 46 (18.33) |  | 8.14 (4.16-16.49) |
| Bacterial vaginosis, n (%) (N=91) | 44 (58.67) | 47 (18.73) |  | 2.47 (1.03-5.80) |
| White blood cells urine dipstick |  |  |  |  |
| ≥ 25, n (%) (N=19) | 3 (4.00) | 16 (6.27) | **0.019** | REF |
| ≥ 50, n (%) (N=45) | 12 (16.00) | 33 (12.94) |  | 0.52 (0.08-2.32) |
| ≥ 75, n (%) (N=70) | 25 (33.33) | 45 (17.65) |  | 0.34 (0.06-1.36) |
| Negative, n (%) (N=196) | 35 (46.67) | 161 (63.14) |  | 0.86 (0.15-3.26) |

| **N=331** | ***Gardnerella piotti* (N=76)** | **No *Gardnerella piotii* (N=255)** | **P-value** | **Odds ratio (95%CI)** |
| --- | --- | --- | --- | --- |
| Mean number of white blood cells on wet mount per field | 10.65 | 8.35 | 0.633 | NA |
| Mean number of epithelial cells on wet mount per field | 25.96 | 26.26 | 0.723 | NA |
| Mean Nugent score | 5.92 | 2.62 | **<0.001** | NA |
| Mean vaginal pH | 6.11 | 5.89 | 0.088 | NA |
| Mean length cervix, cm | 38.57 | 38.30 | 0.942 | NA |
| Mean birthweight, g | 3227.33 | 3230.35 | 0.152 | NA |
| Preterm birth, n (%) (N=30) | 8 (17.78) | 22 (14.01) | 0.634 | 1.32 (0.47-3.41) |
| Low birthweight, n (%) (N=7) | 3 (5.66) | 4 (2.68) | 0.382 | 2.17 (0.31-13.28) |
