# Supplementary material for: Prevalence and clinical correlates of Gardnerella spp., Fannyhessea vaginae, Lactobacillus crispatus and L. iners in pregnant women in Bukavu, Democratic Republic of the Congo
Source: Front Cell Infect Microbiol. 2025 Jan 17;14:1514884. doi: 10.3389/fcimb.2024.1514884 (PMC11782042; doi:10.3389/fcimb.2024.1514884)
Supplement: Supplementary file 3 [file Table3.docx]

**Supplementary Information 3. Univariate associations between Gardnerella swidsinskii and clinical signs and symptoms of mother and baby and pregnancy outcomes.** N, total number of study participants within group; n, number of study participants; OR, odds ratio; CI, confidence interval; NA, not applicable.

| **N=331** | ***Gardnerella swidsinkii* (N=61)** | **No *Gardnerella swidsinkii* (N=270)** | **p-value** | **Odds ratio  (95% CI)** |
| --- | --- | --- | --- | --- |
| Vaginal discharge, n (%) (N=159) | 31 (51.67) | 128 (48.12) | 0.669 | 1.15 (0.63-2.10) |
| Vaginal itching, n (%) (N=136) | 29 (47.54) | 107 (40.07) | 0.315 | 1.35 (0.74-2.46) |
| Dysuria, n (%) (N=86) | 14 (23.73) | 72 (27.17) | 0.629 | 0.83 (0.40-1.66) |
| Burning sensation after sex, n (%) (N=104) | 20 (35.09) | 84 (32.81) | 0.757 | 1.11 (0.57-2.10) |
| Vaginal malodor, n (%) (N=77) | 17 (30.36) | 60 (24.90) | 0.402 | 1.31 (0.65-2.59) |
| Positive whiff test, n (%) (N=31) | 8 (13.33) | 23 (8.58) | 0.326 | 1.64 (0.60-4.06) |
| Anemia, n (%) (N=24) | 4 (6.56) | 20 (7.46) | 1.000 | 0.87 (0.21-2.74) |
| Maternal fever, n (%) (N=37) | 9 (14.75) | 28 (10.65) | 0.374 | 1.45 (0.57-3.40) |
| Uterine contractions, n (%) (N=40) | 7 (14.29) | 33 (13.64) | 0.824 | 1.06 (0.37-2.64) |
| Use of antibiotics 2 weeks  prior to visit, n (%) (N=46) | 6 (9.84) | 40 (14.98) | 0.413 | 0.62 (0.20-1.57) |
| *Trichomonas* on wet mount, n (%) (N=4) | 0 (0.00) | 4 (1.49) | 1.000 | 0.00 (0.00-6.70) |
| *Candida* on wet mount, n (%) (N=91) | 22 (36.07) | 69 (25.75) | 0.114 | 1.62 (0.85-3.04) |
| Infection of baby during  first week of life, n (%) (N=81) | 11 (24.44) | 70 (31.25) | 0.477 | 0.71 (0.31-1.54) |
| Nitrite urine dipstick, n (%) (N=12) | 3 (4.92) | 9 (3.35) | 0.470 | 1.49 (0.25-6.22) |
| State vaginal secretions |  |  |  |  |
| Fine and homogenous, n (%) (N=297) | 54 (88.52) | 243 (90.33) | 0.454 | REF |
| Thick, n (%) (N=16) | 2 (3.28) | 14 (5.20) |  | 0.64 (0.07-2.93) |
| Thick and heterogenous, n (%) (N=17) | 5 (8.20) | 12 (4.46) |  | 1.87 (0.50-6.01) |
| Vulvar state |  |  |  |  |
| Normal, n (%) (N=323) | 59 (96.72) | 264 (98.51) | 0.217 | REF |
| Erythema, n (%) (N=1) | 0 (0.00) | 1 (0.37) |  | 0.00 (0.00-174.69) |
| Postule, n (%) (N=2) | 0 (0.00) | 2 (0.75) |  | 0.00 (0.00-24.13) |
| Leucorrhoea, n (%) (N=3) | 2 (3.28) | 1 (0.37) |  | 8.86 (0.45-527.96) |
| Vaginal microbiome characterization |  |  |  |  |
| Healthy VMB, n (%) (N=176) | 17 (28.33) | 159 (59.77) | **<0.001** | REF |
| Intermediate VMB, n (%) (N=59) | 9 (15.00) | 50 (18.80) |  | 5.54 (2.77-11.45) |
| Bacterial vaginosis, n (%) (N=91) | 34 (56.67) | 57 (21.43) |  | 1.68 (0.62-4.28) |
| White blood cells urine dipstick |  |  |  |  |
| ≥ 25, n (%) (N=19) | 3 (4.92) | 16 (5.95) | 0.180 | REF |
| ≥ 50, n (%) (N=45) | 9 (14.75) | 36 (13.38) |  | 0.75 (0.12-3.58) |
| ≥ 75, n (%) (N=70) | 19 (31.15) | 51 (18.96) |  | 0.51 (0.09-2.07) |
| Negative, n (%) (N=196) | 30 (49.18) | 166 (61.71) |  | 1.04 (0.18-3.96) |

| **N=331** | ***Gardnerella swidsinkii* (N=61)** | **No *Gardnerella swidsinkii* (N=270)** | **p-value** | **Odds ratio  (95% CI)** |
| --- | --- | --- | --- | --- |
| Mean number of white blood cells on wet mount per field | 9.55 | 8.73 | 0.348 | NA |
| Mean number of epithelial cells on wet mount per field | 28.95 | 25.55 | 0.252 | NA |
| Mean Nugent score | 5.57 | 2.89 | **<0.001** | NA |
| Mean vaginal pH | 6.05 | 5.92 | 0.233 | NA |
| Mean length cervix, cm | 38.26 | 38.38 | 0.312 | NA |
| Mean birthweight, g | 3363.78 | 3191.24 | 0.564 | NA |
| Preterm birth, n (%) (N=30) | 4 (8.89) | 26 (16.56) | 0.242 | 0.49 (0.12-1.54) |
| Low birthweight, n (%) (N=7) | 2 (5.00) | 5 (3.09) | 0.627 | 1.65 (0.15-10.55) |
