# Supplementary material for: Prevalence and clinical correlates of Gardnerella spp., Fannyhessea vaginae, Lactobacillus crispatus and L. iners in pregnant women in Bukavu, Democratic Republic of the Congo
Source: Front Cell Infect Microbiol. 2025 Jan 17;14:1514884. doi: 10.3389/fcimb.2024.1514884 (PMC11782042; doi:10.3389/fcimb.2024.1514884)
Supplement: Supplementary file 4 [file Table4.docx]

**Supplementary Information 4. Univariate associations between Gardnerella vaginalis and clinical signs and symptoms of mother and baby and pregnancy outcomes.** N, total number of study participants within group; n, number of study participants; OR, odds ratio; CI, confidence interval; NA, not applicable.

| **N=331** | ***Gardnerella vaginalis* (N=112)** | **No *Gardnerella vaginalis* (N=219)** | **p-value** | **Odds ratio  (95% CI)** |
| --- | --- | --- | --- | --- |
| Vaginal discharge, n (%) (N=159) | 54 (48.21) | 105 (49.07) | 0.908 | 0.97 (0.60-1.57) |
| Vaginal itching, n (%) (N=136) | 45 (40.18) | 91 (42.13) | 0.813 | 0.92 (0.56-1.50) |
| Dysuria, n (%) (N=86) | 24 (22.02) | 62 (28.84) | 0.231 | 0.70 (0.39-1.23) |
| Burning sensation after sex, n (%) (N=104) | 32 (29.91) | 72 (34.95) | 0.380 | 0.79 (0.46-1.35) |
| Vaginal malodor, n (%) (N=77) | 32 (30.77) | 45 (23.32) | 0.168 | 1.46 (0.82-2.57) |
| Positive whiff test, n (%) (N=31) | 18 (16.07) | 13 (6.02) | **0.005** | 2.98 (1.32-6.91) |
| Anemia, n (%) (N=24) | 10 (8.93) | 14 (6.45) | 0.503 | 1.42 (0.54-3.58) |
| Maternal fever, n (%) (N=37) | 11 (9.91) | 26 (12.21) | 0.586 | 0.79 (0.34-1.74) |
| Uterine contractions, n (%) (N=40) | 8 (8.33) | 32 (16.41) | 0.070 | 0.46 (0.18-1.09) |
| Use of antibiotics 2 weeks  prior to visit, n (%) (N=46) | 13 (11.71) | 33 (15.21) | 0.502 | 0.74 (0.34-1.53) |
| *Trichomonas* on wet mount, n (%) (N=4) | 2 (1.80) | 2 (0.92) | 0.606 | 1.98 (0.14-27.62) |
| *Candida* on wet mount, n (%) (N=91) | 24 (21.62) | 67 (30.73) | 0.091 | 0.62 (0.35-1.09) |
| Infection of baby during  first week of life, n (%) (N=81) | 20 (23.26) | 61 (33.33) | 0.117 | 0.61 (0.32-1.12) |
| Nitrite urine dipstick, n (%) (N=12) | 5 (4.46) | 7 (3.21) | 0.550 | 1.41 (0.34-5.29) |
| State vaginal secretions |  |  |  |  |
| Fine and homogenous, n (%) (N=297) | 103 (91.96) | 194 (88.99) | 0.703 | REF |
| Thick, n (%) (N=16) | 5 (4.46) | 11 (5.05) |  | 0.86 (0.23-2.76) |
| Thick and heterogenous, n (%) (N=17) | 4 (3.57) | 13 (5.96) |  | 0.58 (0.13-1.94) |
| Vulvar state |  |  |  |  |
| Normal, n (%) (N=323) | 108 (97.30) | 215 (98.62) | 0.057 | REF |
| Erythema, n (%) (N=1) | 0 (0.00) | 1 (0.46) |  | 0.00 (0.00-77.91) |
| Postule, n (%) (N=2) | 0 (0.00) | 2 (0.92) |  | 0.00 (0.00-10.71) |
| Leucorrhoea, n (%) (N=3) | 3 (2.70) | 0 (0.00) |  | Inf (0.81-inf) |
| Vaginal microbiome characterization |  |  |  |  |
| Healthy VMB, n (%) (N=176) | 30 (27.52) | 146 (67.28) | **<0.001** | REF |
| Intermediate VMB, n (%) (N=59) | 22 (20.18) | 37 (17.05) |  | 8.08 (4.40-15.17) |
| Bacterial vaginosis, n (%) (N=91) | 57 (52.29) | 34 (15.67) |  | 2.88 (1.41-5.85) |
| White blood cells urine dipstick |  |  |  |  |
| ≥ 25, n (%) (N=19) | 8 (7.14) | 11 (5.05) | 0.237 | REF |
| ≥ 50, n (%) (N=45) | 14 (12.50) | 31 (14.22) |  | 1.60 (0.45-5.55) |
| ≥ 75, n (%) (N=70) | 30 (26.79) | 40 (18.35) |  | 0.97 (0.30-3.03) |
| Negative, n (%) (N=196) | 60 (53.57) | 136 (62.39) |  | 1.64 (0.54-4.75) |

| **N=331** | ***Gardnerella vaginalis* (N=112)** | **No *Gardnerella vaginalis* (N=219)** | **p-value** | **Odds ratio  (95% CI)** |
| --- | --- | --- | --- | --- |
| Mean number of white blood cells on wet mount per field | 8.69 | 8.98 | 0.657 | NA |
| Mean number of epithelial cells on wet mount per field | 25.56 | 26.51 | 0.277 | NA |
| Mean Nugent score | 5.47 | 2.33 | **<0.001** | NA |
| Mean vaginal pH | 5.99 | 5.91 | 0.438 | NA |
| Mean length cervix, cm | 39.09 | 37.99 | 0.762 | NA |
| Mean birthweight, g | 3215.49 | 3237.54 | 0.081 | NA |
| Preterm birth, n (%) (N=30) | 8 (10.96) | 22 (17.05) | 0.305 | 0.60 (0.22-1.50) |
| Low birthweight, n (%) (N=7) | 7 (10.29) | 0 (0.00) | **<0.001** | inf (3.03-inf) |
