# Supplementary material for: Prevalence and clinical correlates of Gardnerella spp., Fannyhessea vaginae, Lactobacillus crispatus and L. iners in pregnant women in Bukavu, Democratic Republic of the Congo
Source: Front Cell Infect Microbiol. 2025 Jan 17;14:1514884. doi: 10.3389/fcimb.2024.1514884 (PMC11782042; doi:10.3389/fcimb.2024.1514884)
Supplement: Supplementary file 5 [file Table5.docx]

**Supplementary Information 5. Univariate associations between Fannyhessea vaginae and clinical signs and symptoms of mother and baby and pregnancy outcomes.** N, total number of study participants within group; n, number of study participants; OR, odds ratio; CI, confidence interval; NA, not applicable.

| **N=331** | ***Fannyhessea vaginae* (N=120)** | **No *Fannyhessea vaginae* (N=211)** | **p-value** | **Odds ratio  (95% CI)** |
| --- | --- | --- | --- | --- |
| Vaginal discharge, n (%) (N=159) | 60 (50.85) | 99 (47.60) | 0.6448 | 1.14 (0.71-1.84) |
| Vaginal itching, n (%) (N=136) | 52 (43.70) | 84 (40.19) | 0.5612 | 1.15 (0.71-1.87) |
| Dysuria, n (%) (N=86) | 32 (27.35) | 54 (26.09) | 0.7951 | 1.07 (0.62-1.83) |
| Burning sensation after sex, n (%) (N=104) | 41 (35.65) | 63 (31.82) | 0.5342 | 1.19 (0.71-1.98) |
| Vaginal malodor, n (%) (N=77) | 32 (29.91) | 45 (23.68) | 0.2705 | 1.37 (0.78-2.42) |
| Positive whiff test, n (%) (N=31) | 20 (16.95) | 11 (5.24) | **0.007** | 3.68 (1.61-8.85) |
| Anemia, n (%) (N=24) | 11 (9.32) | 13 (6.16) | 0.3766 | 1.56 (0.61-3.93) |
| Maternal fever, n (%) (N=37) | 14 (12.07) | 23 (11.06) | 0.8558 | 1.10 (0.50-2.35) |
| Uterine contractions, n (%) (N=40) | 9 (8.57) | 31 (16.67) | 0.0751 | 0.47 (0.19-1.07) |
| Use of antibiotics 2 weeks  prior to visit, n (%) (N=46) | 11 (9.32) | 35 (16.67) | 0.0703 | 0.51 (0.23-1.09) |
| *Trichomonas* on wet mount, n (%) (N=4) | 2 (1.69) | 2 (0.95) | 0.62 | 1.80 (0.13-25.11) |
| *Candida* on wet mount, n (%) (N=91) | 34 (28.81) | 57 (27.01) | 0.7973 | 1.09 (0.64-1.85) |
| Infection of baby during  first week of life, n (%) (N=81) | 25 (26.04) | 56 (32.37) | 0.332 | 0.74 (0.40-1.32) |
| Nitrite urine dipstick, n (%) (N=12) | 6 (5.04) | 6 (2.84) | 0.3626 | 1.81 (0.47-6.95) |
| State vaginal secretions |  |  |  |  |
| Fine and homogenous, n (%) (N=297) | 105 (88.24) | 192 (91.00) | 0.3374 | REF |
| Thick, n (%) (N=16) | 5 (4.20) | 11 (5.21) |  | 0.83 (0.22-2.68) |
| Thick and heterogenous, n (%) (N=17) | 9 (7.56) | 8 (3.79) |  | 2.05 (0.68-6.31) |
| Vulvar state |  |  |  |  |
| Normal, n (%) (N=323) | 115 (97.46) | 208 (98.58) | 0.2044 | REF |
| Erythema, n (%) (N=1) | 1 (0.85) | 0 (0.00) |  | Inf (0.05-inf) |
| Postule, n (%) (N=2) | 0 (0.00) | 2 (0.95) |  | 0.00 (0.00-9.73) |
| Leucorrhoea, n (%) (N=3) | 2 (1.69) | 1 (0.47) |  | 3.60 (0.19-214.13) |
| Vaginal microbiome characterization |  |  |  |  |
| Healthy VMB, n (%) (N=176) | 31 (26.27) | 145 (69.71) | **<0.001** | REF |
| Intermediate VMB, n (%) (N=59) | 21 (17.80) | 38 (18.27) |  | 12.19 (6.50-23.61) |
| Bacterial vaginosis, n (%) (N=91) | 66 (55.93) | 25 (12.02) |  | 2.57 (1.26-5.23) |
| White blood cells urine dipstick |  |  |  |  |
| ≥ 25, n (%) (N=19) | 7 (5.88) | 12 (5.69) | **0.017** | REF |
| ≥ 50, n (%) (N=45) | 19 (15.97) | 26 (12.32) |  | 0.80 (0.22-2.72) |
| ≥ 75, n (%) (N=70) | 35 (29.41) | 35 (16.59) |  | 0.59 (0.17-1.84) |
| Negative, n (%) (N=196) | 58 (48.74) | 138 (65.40) |  | 1.39 (0.44-4.05) |

| **N=331** | ***Fannyhessea vaginae* (N=120)** | **No *Fannyhessea vaginae* (N=211)** | **p-value** | **Odds ratio  (95% CI)** |
| --- | --- | --- | --- | --- |
| Mean number of white blood cells on wet mount per field | 9.82 | 8.35 | **0.045** | NA |
| Mean number of epithelial cells on wet mount per field | 27.64 | 25.38 | 0.1754 | NA |
| Mean Nugent score | 5.56 | 2.14 | **<0.001** | NA |
| Mean vaginal pH | 6.07 | 5.87 | **0.001** | NA |
| Mean length cervix, cm | 38.77 | 38.13 | 0.958 | NA |
| Mean birthweight, g | 3204.24 | 3243.77 | 0.603 | NA |
| Preterm birth, n (%) (N=30) | 9 (12.50) | 21 (16.15) | 0.541 | 0.74 (0.28-1.82) |
| Low birthweight, n (%) (N=7) | 6 (7.41) | 1 (0.83) | **0.017** | 9.50  (1.12-444.16) |
