# Supplementary material for: Prevalence and clinical correlates of Gardnerella spp., Fannyhessea vaginae, Lactobacillus crispatus and L. iners in pregnant women in Bukavu, Democratic Republic of the Congo
Source: Front Cell Infect Microbiol. 2025 Jan 17;14:1514884. doi: 10.3389/fcimb.2024.1514884 (PMC11782042; doi:10.3389/fcimb.2024.1514884)
Supplement: Supplementary file 6 [file Table6.docx]

**Supplementary Information 6. Univariate associations between Lactobacillus crispatus and clinical signs and symptoms of mother and baby and pregnancy outcomes.** N, total number of study participants within group; n, number of study participants; OR, odds ratio; CI, confidence interval; NA, not applicable.

| **N=331** | ***Lactobacillus crispatus* (N=136)** | **No *Lactobacillus crispatus* (N=195)** | **p-value** | **Odds ratio  (95% CI)** |
| --- | --- | --- | --- | --- |
| Vaginal discharge, n (%) (N=159) | 63 (46.67) | 96 (50.26) | 0.574 | 0.87 (0.54-1.38) |
| Vaginal itching, n (%) (N=136) | 49 (36.30) | 87 (45.08) | 0.139 | 0.69 (0.43-1.12) |
| Dysuria, n (%) (N=86) | 33 (24.81) | 53 (27.75) | 0.610 | 0.86 (0.50-1.46) |
| Burning sensation after sex, n (%) (N=104) | 38 (29.23) | 66 (36.07) | 0.225 | 0.73 (0.44-1.22) |
| Vaginal malodor, n (%) (N=77) | 29 (24.79) | 48 (26.67) | 0.787 | 0.91 (0.51-1.59) |
| Positive whiff test, n (%) (N=31) | 8 (5.93) | 23 (11.92) | 0.084 | 0.47 (0.17-1.12) |
| Anemia, n (%) (N=24) | 13 (9.56) | 11 (5.70) | 0.202 | 1.75 (0.70-4.46) |
| Maternal fever, n (%) (N=37) | 13 (9.70) | 24 (12.63) | 0.480 | 0.74 (0.33-1.59) |
| Uterine contractions, n (%) (N=40) | 18 (14.75) | 22 (13.02) | 0.731 | 1.16 (0.55-2.39) |
| Use of antibiotics 2 weeks  prior to visit, n (%) (N=46) | 17 (12.59) | 29 (15.03) | 0.629 | 0.82 (0.40-1.62) |
| *Trichomonas* on wet mount, n (%) (N=4) | 1 (0.74) | 3 (1.55) | 0.647 | 0.48 (0.01-6.00) |
| *Candida* on wet mount, n (%) (N=91) | 33 (24.44) | 58 (29.90) | 0.317 | 0.76 (0.44-1.28) |
| Infection of baby during  first week of life, n (%) (N=81) | 37 (33.04) | 44 (28.03) | 0.419 | 1.27 (0.72-2.21) |
| Nitrite urine dipstick, n (%) (N=12) | 5 (3.68) | 7 (3.61) | 1.000 | 1.02 (0.25-3.82) |
| State vaginal secretions |  |  |  |  |
| Fine and homogenous, n (%) (N=297) | 128 (94.12) | 169 (87.11) | 0.090 | REF |
| Thick, n (%) (N=16) | 3 (2.21) | 13 (6.70) |  | 0.31 (0.05-1.14) |
| Thick and heterogenous, n (%) (N=17) | 5 (3.68) | 12 (6.19) |  | 0.55 (0.15-1.73) |
| Vulvar state |  |  |  |  |
| Normal, n (%) (N=323) | 133 (98.52) | 190 (97.94) | 0.293 | REF |
| Erythema, n (%) (N=1) | 1 (0.74) | 0 (0.00) |  | Inf (0.04-inf) |
| Postule, n (%) (N=2) | 1 (0.74) | 1 (0.52) |  | 1.43 (0.02-112.62) |
| Leucorrhoea, n (%) (N=3) | 0 (0.00) | 3 (1.55) |  | 0.00 (0.00-3.51) |
| Vaginal microbiome characterization |  |  |  |  |
| Healthy VMB, n (%) (N=176) | 90 (66.67) | 86 (45.03) | **<0.001** | REF |
| Intermediate VMB, n (%) (N=59) | 22 (16.30) | 37 (19.37) |  | 0.32 (0.18-0.58) |
| Bacterial vaginosis, n (%) (N=91) | 23 (17.04) | 68 (35.60) |  | 0.57 (0.29-1.08) |
| White blood cells urine dipstick |  |  |  |  |
| ≥ 25, n (%) (N=19) | 9 (6.62) | 10 (5.15) | 0.390 | REF |
| ≥ 50, n (%) (N=45) | 14 (10.29) | 31 (15.98) |  | 1.97 (0.57-6.85) |
| ≥ 75, n (%) (N=70) | 27 (19.85) | 43 (22.16) |  | 1.43 (0.45-4.49) |
| Negative, n (%) (N=196) | 86 (63.24) | 110 (56.70) |  | 1.15 (0.39-3.31) |

| **N=331** | ***Lactobacillus crispatus* (N=136)** | **No *Lactobacillus crispatus* (N=195)** | **p-value** | **Odds ratio  (95% CI)** |
| --- | --- | --- | --- | --- |
| Mean number of white blood cells on wet mount per field | 7.98 | 9.51 | 0.623 | NA |
| Mean number of epithelial cells on wet mount per field | 26.43 | 26.02 | 0.912 | NA |
| Mean Nugent score | 2.43 | 4.05 | **<0.001** | NA |
| Mean vaginal pH | 5.88 | 5.98 | **0.043** | NA |
| Mean length cervix, cm | 38.79 | 38.06 | 0.653 | NA |
| Mean birthweight, g | 3282.74 | 3191.91 | 0.157 | NA |
| Preterm birth, n (%) (N=30) | 11 (13.25) | 19 (15.97) | 0.689 | 0.80 (0.32-1.91) |
| Low birthweight, n (%) (N=7) | 4 (5.00) | 3 (2.46) | 0.438 | 2.08 (0.34-14.59) |
