# Supplementary material for: Prevalence and clinical correlates of Gardnerella spp., Fannyhessea vaginae, Lactobacillus crispatus and L. iners in pregnant women in Bukavu, Democratic Republic of the Congo
Source: Front Cell Infect Microbiol. 2025 Jan 17;14:1514884. doi: 10.3389/fcimb.2024.1514884 (PMC11782042; doi:10.3389/fcimb.2024.1514884)
Supplement: Supplementary file 7 [file Table7.docx]

**Supplementary Information 7. Univariate associations between Lactobacillus iners and clinical signs and symptoms of mother and baby and pregnancy outcomes.** N, total number of study participants within group; n, number of study participants; OR, odds ratio; CI, confidence interval; NA, not applicable.

| **N=331** | ***Lactobacillus iners* (N=251)** | **No *Lactobacillus iners* (N=76)** | **p-value** | **Odds ratio  (95% CI)** |
| --- | --- | --- | --- | --- |
| Vaginal discharge, n (%) (N=159) | 116 (46.77) | 41 (55.41) | 0.233 | 0.71 (0.40-1.23) |
| Vaginal itching, n (%) (N=136) | 104 (41.77) | 31 (41.33) | 1.000 | 1.02 (0.58-1.79) |
| Dysuria, n (%) (N=86) | 67 (27.13) | 19 (26.03) | 1.000 | 1.06 (0.57-2.03) |
| Burning sensation after sex, n (%) (N=104) | 78 (33.05) | 25 (34.25) | 0.887 | 0.95 (0.53-1.73) |
| Vaginal malodor, n (%) (N=77) | 55 (24.66) | 22 (30.56) | 0.355 | 0.74 (0.40-1.41) |
| Positive whiff test, n (%) (N=31) | 26 (10.44) | 5 (6.67) | 0.380 | 1.63 (0.59-5.64) |
| Anemia, n (%) (N=24) | 18 (7.20) | 6 (8.00) | 0.803 | 0.89 (0.32-2.86) |
| Maternal fever, n (%) (N=37) | 34 (13.77) | 3 (4.11) | **0.022** | 3.71 (1.11-19.47) |
| Uterine contractions, n (%) (N=40) | 33 (14.93) | 7 (10.61) | 0.425 | 1.48 (0.60-4.17) |
| Use of antibiotics 2 weeks  prior to visit, n (%) (N=46) | 31 (12.45) | 14 (18.67) | 0.184 | 0.62 (0.30-1.35) |
| *Trichomonas* on wet mount, n (%) (N=4) | 4 (1.60) | 0 (0.00) | 0.577 | inf (0.20-inf) |
| *Candida* on wet mount, n (%) (N=91) | 77 (30.80) | 14 (18.67) | **0.041** | 1.94 (1.00-3.98) |
| Infection of baby during  first week of life, n (%) (N=81) | 62 (29.95) | 17 (29.31) | 1.000 | 1.03 (0.53-2.09) |
| Nitrite urine dipstick, n (%) (N=12) | 11 (4.38) | 1 (1.33) | 0.309 | 3.38 (0.48-147.85) |
| State vaginal secretions |  |  |  |  |
| Fine and homogenous, n (%) (N=297) | 222 (88.45) | 71 (94.67) | 0.201 | REF |
| Thick, n (%) (N=16) | 13 (5.18) | 3 (4.00) |  | 1.38 (0.37-7.79) |
| Thick and heterogenous, n (%) (N=17) | 16 (6.37) | 1 (1.33) |  | 5.10 (0.77-217.38) |
| Vulvar state |  |  |  |  |
| Normal, n (%) (N=323) | 244 (97.60) | 75 (100) | 1.000 | REF |
| Erythema, n (%) (N=1) | 1 (0.40) | 0 (0.00) |  | Inf (0.01-inf) |
| Postule, n (%) (N=2) | 2 (0.80) | 0 (0.00) |  | Inf (0.06-inf) |
| Leucorrhoea, n (%) (N=3) | 3 (1.20) | 0 (0.00) |  | Inf (0.12-inf) |
| Vaginal microbiome characterization |  |  |  |  |
| Healthy VMB, n (%) (N=176) | 141 (56.85) | 33 (44.59) | 0.107 | REF |
| Intermediate VMB, n (%) (N=59) | 40 (16.13) | 19 (25.68) |  | 0.71 (0.37-1.39) |
| Bacterial vaginosis, n (%) (N=91) | 67 (27.02) | 22 (29.73) |  | 0.49 (0.24-1.02) |
| White blood cells urine dipstick |  |  |  |  |
| ≥ 25, n (%) (N=19) | 15 (5.98) | 4 (5.33) | 0.208 | REF |
| ≥ 50, n (%) (N=45) | 40 (15.94) | 5 (6.67) |  | 0.47 (0.09-2.73) |
| ≥ 75, n (%) (N=70) | 52 (20.72) | 17 (22.67) |  | 1.22 (0.33-5.75) |
| Negative, n (%) (N=196) | 144 (57.37) | 49 (65.33) |  | 1.27 (0.38-5.53) |

| **N=331** | ***Lactobacillus iners* (N=251)** | **No *Lactobacillus iners* (N=76)** | **p-value** | **Odds ratio  (95% CI)** |
| --- | --- | --- | --- | --- |
| Mean number of white blood cells on wet mount per field | 9.33 | 7.55 | 0.072 | NA |
| Mean number of epithelial cells on wet mount per field | 27.01 | 23.80 | 0.068 | NA |
| Mean Nugent score | 3.24 | 3.82 | 0.627 | NA |
| Mean vaginal pH | 5.97 | 5.82 | 0.681 | NA |
| Mean length cervix, cm | 38.22 | 38.88 | 0.705 | NA |
| Mean birthweight, g | 3201.03 | 3278.61 | 0.403 | NA |
| Preterm birth, n (%) (N=30) | 27 (18.37) | 3 (5.66) | **0.026** | 3.73 (1.07-20.08) |
| Low birthweight, n (%) (N=7) | 4 (2.65) | 3 (6.38) | 0.360 | 0.40 (0.07-2.84) |
