# Supplementary material for: Prevalence and clinical correlates of Gardnerella spp., Fannyhessea vaginae, Lactobacillus crispatus and L. iners in pregnant women in Bukavu, Democratic Republic of the Congo
Source: Front Cell Infect Microbiol. 2025 Jan 17;14:1514884. doi: 10.3389/fcimb.2024.1514884 (PMC11782042; doi:10.3389/fcimb.2024.1514884)
Supplement: Supplementary file 8 [file Table8.docx]

**Supplementary Information 8. Univariate associations between clusters and clinical signs and symptoms of mother and baby and pregnancy outcomes.** N, total number of study participants within group; n, number of study participants; OR, odds ratio; CI, confidence interval.

| **N=331** | **Cluster 1 (N=26)** | **Cluster 2 (N=45)** | **Cluster 3 (N=42)** | **Cluster 4 (N=32)** | **Cluster 5 (N=79)** | **Cluster 6 (N=87)** | **p-value** |
| --- | --- | --- | --- | --- | --- | --- | --- |
| Vaginal discharge, n (%) (N=159) | 17 (68.00) | 20 (45.45) | 20 (47.62) | 18 (58.06) | 34 (43.04) | 39 (45.35) | 0.274 |
| Vaginal itching, n (%) (N=136) | 10 (40.00) | 22 (48.89) | 17 (40.48) | 12 (37.50) | 38 (48.72) | 28 (32.56) | 0.327 |
| Dysuria, n (%) (N=86) | 8 (33.33) | 8 (18.18) | 8 (20.00) | 8 (25.00) | 28 (35.90) | 23 (26.74) | 0.284 |
| Burning sensation after sex, n (%) (N=104) | 7 (29.17) | 13 (30.95) | 11 (27.50) | 16 (53.33) | 28 (37.33) | 24 (28.92) | 0.207 |
| Vaginal malodor, n (%) (N=77) | 5 (20.83) | 13 (32.50) | 9 (23.68) | 11 (36.67) | 15 (20.83) | 18 (24.32) | 0.529 |
| Positive whiff test, n (%) (N=31) | 2 (8.00) | 6 (13.64) | 8 (19.05) | 6 (18.75) | 5 (6.41) | 2 (2.30) | **0.005** |
| Anemia, n (%) (N=24) | 1 (4.00) | 4 (8.89) | 5 (11.90) | 1 (3.12) | 3 (3.85) | 10 (11.49) | 0.338 |
| Maternal fever, n (%) (N=37) | 0 (0.00) | 8 (17.78) | 7 (17.07) | 6 (18.75) | 9 (11.84) | 5 (5.81) | **0.040** |
| Uterine contractions, n (%) (N=40) | 0 (0.00) | 7 (18.42) | 4 (10.26) | 4 (14.29) | 11 (15.71) | 12 (15.58) | 0.406 |
| Use of antibiotics 2 weeks  prior to visit, n (%) (N=46) | 4 (16.00) | 5 (11.11) | 5 (12.20) | 4 (12.50) | 14 (17.95) | 12 (13.79) | 0.925 |
| *Trichomonas* on wet mount, n (%) (N=4) | 0 (0.00) | 0 (0.00) | 2 (4.88) | 0 (0.00) | 2 (2.53) | 0 (0.00) | 0.187 |
| *Candida* on wet mount, n (%) (N=91) | 4 (16.00) | 19 (42.22) | 7 (17.07) | 12 (37.70) | 25 (31.65) | 20 (22.99) | **0.038** |
| Infection of baby during  first week of life, n (%) (N=81) | 5 (26.32) | 10 (28.57) | 6 (17.14) | 6 (24.00) | 23 (36.51) | 28 (37.33) | 0.284 |
| Nitrite urine dipstick, n (%) (N=12) | 0 (0.00) | 3 (6.67) | 3 (7.14) | 0 (0.00) | 4 (5.06) | 1 (1.15) | 0.212 |
| State vaginal secretions |  |  |  |  |  |  |  |
| Fine and homogenous, n (%) (N=297) | 25 (100) | 38 (84.44) | 37 (88.10) | 28 (87.50) | 69 (87.34) | 84 (96.55) | 0.158 |
| Thick, n (%) (N=16) | 0 (0.00) | 2 (4.44) | 3 (7.14) | 3 (9.38) | 4 (5.06) | 1 (1.15) |  |
| Thick and heterogenous, n (%) (N=17) | 0 (0.00) | 5 (11.11) | 2 (4.76) | 1 (3.12) | 6 (7.59) | 2 (2.30) |  |
| Vulvar state |  |  |  |  |  |  |  |
| Normal, n (%) (N=323) | 25 (100) | 43 (95.56) | 40 (97.56) | 32 (100) | 78 (98.73) | 85 (97.70) | 0.560 |
| Erythema, n (%) (N=1) | 0 (0.00) | 0 (0.00) | 0 (0.00) | 0 (0.00) | 0 (0.00) | 1 (1.15) |  |
| Postule, n (%) (N=2) | 0 (0.00) | 0 (0.00) | 0 (0.00) | 0 (0.00) | 1 (1.27) | 1 (1.15) |  |
| Leucorrhoea, n (%) (N=3) | 0 (0.00) | 2 (4.44) | 1 (2.44) | 0 (0.00) | 0 (0.00) | 0 (0.00) |  |

| **N=331** | **Cluster 1 (N=26)** | **Cluster 2 (N=45)** | **Cluster 3 (N=42)** | **Cluster 4 (N=32)** | **Cluster 5 (N=79)** | **Cluster 6 (N=87)** | **p-value** |
| --- | --- | --- | --- | --- | --- | --- | --- |
| Vaginal microbiome characterization |  |  |  |  |  |  |  |
| Healthy VMB, n (%) (N=176) | 6 (24.00) | 12 (27.27) | 12 (29.27) | 7 (21.88) | 59 (74.68) | 70 (81.40) | **<0.001** |
| Intermediate VMB, n (%) (N=59) | 8 (32.00) | 5 (11.36) | 8 (19.51) | 8 (25.00) | 15 (18.99) | 11 (12.79) |  |
| Bacterial vaginosis, n (%) (N=91) | 11 (44.00) | 27 (61.36) | 21 (51.22) | 17 (53.12) | 5 (6.33) | 5 (5.81) |  |
| White blood cells urine dipstick |  |  |  |  |  |  |  |
| ≥ 25, n (%) (N=19) | 3 (12.00) | 2 (4.44) | 3 (7.14) | 0 (0.00) | 5 (6.33) | 6 (6.90) | 0.536 |
| ≥ 50, n (%) (N=45) | 2 (8.00) | 8 (17.78) | 7 (16.67) | 6 (18.75) | 14 (17.72) | 8 (9.20) |  |
| ≥ 75, n (%) (N=70) | 6 (24.00) | 13 (28.89) | 9 (21.43) | 9 (28.12) | 15 (18.99) | 14 (16.09) |  |
| Negative, n (%) (N=196) | 14 (56.00) | 22 (48.89) | 23 (54.76) | 17 (53.12) | 45 (56.96) | 59 (67.82) |  |
| Mean number of white blood cells on wet mount per field | 7.00 | 11.27 | 8.48 | 9.06 | 10.29 | 7.29 | 0.080 |
| Mean number of epithelial cells on wet mount per field | 27.60 | 28.60 | 25.00 | 30.10 | 26.40 | 24.50 | 0.194 |
| Mean Nugent score | 5.36 | 5.70 | 5.51 | 5.47 | 1.85 | 1.28 | **<0.001** |
| Mean vaginal pH | 5.88 | 6.18 | 6.02 | 6.00 | 5.90 | 5.84 | 0.516 |
| Mean length cervix, cm | 37.20 | 38.70 | 40.00 | 37.80 | 37.30 | 38.70 | 0.864 |
| Mean birthweight, g | 3234.67 | 3394.12 | 3143.64 | 3127.61 | 3053.41 | 3283.46 | 0.477 |
| Preterm birth, n (%) (N=30) | 0 (0.00) | 4 (11.76) | 3 (13.04) | 3 (13.04) | 9 (17.65) | 10 (24.39) | 0.332 |
| Low birthweight, n (%) (N=7) | 0 (0.00) | 0 (0.00) | 3 (13.64) | 1 (4.35) | 1 (2.44) | 2 (3.85) | 0.208 |
